# Investigating double-diffusive natural convection in a sloped dual-layered homogenous porous-fluid square cavity

**DOI:** 10.1038/s41598-024-57395-2

**Published:** 2024-03-26

**Authors:** Bahram Jalili, Majdeddin Emad, Emad Hasani Malekshah, Payam Jalili, Ali Akgül, Murad Khan Hassani

**Affiliations:** 1 Department of Mechanical Engineering, North Tehran Branch, Islamic Azad University, Tehran, Iran; 2 Department of Power Engineering and Turbomachinery, Silesian University of Technology, 44-10 0, Gliwice, Poland; 3 Department of Computer Science and Mathematics, Lebanese American University, Beirut, Lebanon; 4 Department of Mathematics, Art and Science Faculty, Siirt University, 56100 Siirt, Turkey; 5 Department of Mathematics, Ghazni University, Ghazni, Afghanistan

**Keywords:** Convective flow, Natural convection, Porous medium, Double diffusion, Finite element method, Biological techniques, Biophysics

## Abstract

This article investigates natural convection with double-diffusive properties numerically in a vertical bi-layered square enclosure. The cavity has two parts: one part is an isotropic and homogeneous porous along the wall, and an adjacent part is an aqueous fluid. Adiabatic, impermeable horizontal walls and constant and uniform temperatures and concentrations on other walls are maintained. To solve the governing equations, the finite element method (FEM) employed and predicted results shows the impact of typical elements of convection on double diffusion, namely the porosity thickness, cavity rotation angle, and thermal conductivity ratio. Different Darcy and Rayleigh numbers effects on heat transfer conditions were investigated, and the Nusselt number in the border of two layers was obtained. The expected results, presented as temperature field (isothermal lines) and velocity behavior in X and Y directions, show the different effects of the aforementioned parameters on double diffusion convective heat transfer. Also results show that with the increase in the thickness of the porous layer, the Nusselt number decreases, but at a thickness higher than 0.8, we will see an increase in the Nusselt number. Increasing the thermal conductivity ratio in values less than one leads to a decrease in the average Nusselt number, and by increasing that parameter from 1 to 10, the Nusselt values increase. A higher rotational angle of the cavity reduces the thermosolutal convective heat transfer, and increasing the Rayleigh and Darcy numbers, increases Nusselt. These results confirm that the findings obtained from the Finite Element Method (FEM), which is the main idea of this research, are in good agreement with previous studies that have been done with other numerical methods.

## Introduction

When a multifactor liquid (a liquid that carries other types of material components rather than the main liquid) withstands the change in density (which is caused by differences in temperature or species concentrations), the gravity-produced buoyancy forces are induced in the fluid, then convection to be expected. The expansion of industrial processes, which include heat transfer and mass diffusion in a binary fluid, encouraged many researchers to do experimental research, theory, and numerical studies. In many engineering industries, such as alloy solidification processes, as well as in geophysics, oceanography, insulations, etc., and subjects like diffusion of pollutants in soil, drying of agricultural products and pharmaceuticals, diffusion of the radioactive waste underground deposits, energy storage, this issue has been taken into consideration. In recent years, numerical investigations of the dual diffusion free convection mass and heat transfer of the cavities have been proposed by many scholars. Hart et al.^1^ were the pioneers who studied the phenomenon of double diffusion numerically for laminar flow regimes through surfaces and columns.

Bejan^2^ proposed the first principal scales of heat and mass transfer coefficients. He accomplished the numerical analysis of a closed cavity, which included Newman boundary condition vertical walls. In 1985, he conducted and published basic research on free convection in a cavity with a rectangular form and the side’s heat transfer, which is the fundamental research to continue investigating this type of heat transfer. Haghighat et al.^3^ studied steady-state convection with thermosolutal property in an air square enclosure. The changes in concentration and temperature on the horizontal surface was considered, and the buoyancy force of solubility impact on heat transfer was investigated. A correlation was obtained dependent on both Sherwood and Nusselt numbers. Gobin and Bennacer^4^ investigated free heat transfer in a binary fluid layer. Stability analysis for the unbounded layer had shown that inertia prevails at “moderate” Grashof numbers. They found the relation of Lewis and critical Rayleigh number. An investigation was made in a porous chamber by Goya et al.^5^, which used the Darcy–Brinkman model. They had shown that the thermal analysis was in good contrast with the scale analysis, but the boundary layer analysis was not a suitable way for predicting the heat transfer correct scales in a similar field. Bennacer et al.^6^ managed a numerical investigation in a vertical annular porous material. Darcy’s extended Brinkman model was used to establish the non-slip condition over the solid wall and found that the impacts of radius ratio and Darcy number on both average Nusselt and Sherwood numbers were very significant. Bennacer et al.^7^ conducted a study on anisotropic porous. Correlations for Sherwood and Nusselt numbers were presented, which include the properties of anisotropic porous material. Later, they investigated a closed chamber that includes two layers of porous material that are completely parallel on both sides with an air space between them^8^. The obtained result showed that when the fluid entered the porous material, Nusselt numbers were a function of anisotropy, and the heat transfer rate decreased. Costa^9^ did the mentioned subject on a parallelogram enclosure. The general result of this research was a rise in thermal transfer influenced by augmentation in the ratio of Buoyancy and Rayleigh. However, encouraging heat transfer was impacted by rotational angle and aspect ratio. This subject was investigated by Gobin et al.^10^. It was found that the specific behavior of the flow structure was the result of flow penetration inside the porous part and buoyancy forces. Bahloul^11^ studied a rectangular cavity. For large Rayleigh numbers and based on numerical results, an approximation for the boundary layer regime model was received. He presented a simplified template for the stratification parameter. A numerical investigation for closed porous chamber with heating element and a salting element on one side conducted by Zhao et al.^12^.

Teamah^13^ studied a chamber with insulated above and lower walls. The chamber area was influenced by a heat source plus a magnetic field. Chamkha and Al-Mudhaf^14^ researched rectangular cavities with homogeneous porous media under the impact of a heat source and sink. They had shown that decreasing of the Darcy number, decreased the Sherwood and Nusselt numbers alongside fluid circulation in the chamber. Baytash et al.’s^15^ research was about a fluid-saturated porous inside a closed chamber using the non-Darcy model. The most important result of this research was that if the intersection of two walls of fluid and porous material is not horizontal but has a step, the convective heat transfer will change dramatically. Nouanegue et al.^16^ investigated conduction plus radiation in an enclosure. It was found that the opposition of three types of heat transfer had great impacts, and the impact of surface radiation on “free convection” cannot be ignored. Khanafer et al.^17^ performed some studies on a cavity with a sinusoidal wall in 2008. They had shown encouragement of convection with the raising of Rayleigh numbers. Akbal et al.^18^ worked on a porous cavity with variable porosity and partially permeable walls using Darcy’s law. The principal findings of this study were about concentration and temperature gradients, which in all cases were greater according to the homogenous porosity. In the continuation of the research, an annular cylinder with porous investigated by Bennacer et al.^19^. With the constant temperature differences, impermeable and adiabatic horizontal surfaces, and considering the “Soret effect”, it was demonstrated that the larger thermal gradients were more possible in the cylindrical annulus. Al-Farhani et al.^20^ showed that when the rotational angle and aspect ratio rise, Sherwood and Nusselt numbers decrease. Bennacer et al.^21^ investigated the impact of sloped and bi-layered porous enclosures that were uniformly heated and linearly cooled the left and right side walls. Other walls were insulated respectively. An optimal tilt angle, which leads to a maximum value of heat transfer, was demonstrated. Teamah et al.^22^ discussed numerical simulations of rectangular enclosures with a slope versus the impact of heat source and magnetism. They had obtained that for lower Rayleigh number, conduction prevails and with augmentation and total heat transfer increased. Hadidi^23^ conducted a numerical study for a chamber made of two layers of porous material. This research showed that layers of porous media have a remarkable effect on the flow structure besides its heat transfer. Jagadeesha et al.^24^ also performed double diffusion in an angled parallelogram containing a porous material. The important result of this experiment showed the dependency of heat transfer rates and mass transfer, Darcy and Rayleigh numbers and rotational angle. Where the Rayleigh number and rotation angle increased, growth of the convection efficiency was visible. El-Moutaouakil et al.^25^ reported the analytical answer according to the parallel flow approximation of an angular enclosure with volumetric cooling liquid alongside heat generation. They reported that the analytical and numerical solution agreement is perfect for inclination angles between 15 and 165°. Bhardwaj et al.^26^ surveyed the impact of corrugated walls and consistent heating on entropy production and natural convection in a closed collection of porous material bottom, and the left wall was heated. They found that for low values of Rayleigh, convection was significant, while heat transfer was under the influence of conduction for higher values of Rayleigh.

Meerali et al.^27^ surveyed a chamber in the presence of a heating element filled with several porous layers plus nanofluids. The cavity contained two layers of nanofluids, “water and TiO2”, on both sides and a porous part between them. It was observed that the stream function maximum absolute value increases as the porous material width decreases. Siavashi et al.^28^ conducted a two-phase fluid for a closed square chamber with a fluid inside non-Darcy porous material under the heat source and a solvent influence. The problem was solved with the finite volume method by employing the Darcy–Brinkman–Forkheimer model. Hu et al.^29^ studied an enclosure that included a part of porous material and another part of solid material with heat generation. This research showed that the forces of thermal Buoyancy and solubility help each other, and the negative sign of the buoyancy coefficient only affects fluid orientation. Hadidi and Bennacer^30^ investigated a 3D heat transfer of a two-layer cube with a temperature difference and thickness in the walls, a double diffusion type of convection. 2 or 3 flow regimes were observed, which had a dependency on the Darcy numbers. Venkatadri et al.^31^ considered a model with a sloped upper wall, adiabatic right and left walls and a constant temperature bottom wall. What was obtained from the calculations showed a relation of low Rayleigh numbers (1000) and very low heat and mass transfer rate. Mehryan et al.^32^ investigated a trapezoidal chamber separated by an elastic partition. They found that the rate of heat transfer in a square chamber is 15% bigger than a trapezoid one with side angles of 30 degrees. It was by Hu et al.^33^ in their research, the effects of Soret and Dufour were examined inside the chamber. Soret number had soft effects on heat and boot’s moisture transfer. Dufour growth prevented heat and diminished the moisture transfer rate. Hadidi and Bennacer, among other researchers^34^, did a finite volume method study of a rotational bi-layered cavity convection in which a vertically porous filled part was in conjunction with an aqueous solution. They found thermosolutal parameters were dependent on Sherwood and Nusselt and porous material effects on the hydrothermal behavior of the chamber. Abdelraheem et al.^35^ studied NEPCM (Nano-Enhanced Phase Change Materials) Magneto Hydro Dynamic (MHD) convection influenced by thermal radiation. The suspension was in a horizontal embedded high-temperature crescents wavy porous cavity. Jalili et al.^36^ surveyed a solution of MHD heat transfer of nanofluid for a Cassini oval containing circular porous material with the effects of the buoyancy and Lorentz forces. Combination impacts of various Darcy and Rayleigh numbers on free convection of 2 vertical hot porous shafts with several diameters values numerically studied by Shruti et al.^37^ in 2023. Javed et al.^38^ investigated a convection and entropy generation of a square enclosure divided by corrugated porous parts. They found the better configuration for heat transfer was the case where the partition was closer to the heated wall, and the highest domain and corrugation frequency ensured the highest heat transfer. Abdollahi et al.^39^ conducted computer-aided simulations for a Cu: AlOOH/water inside a micro duct heating sump using a porous material technique, which was solved with FEM and AGM (Akbari-Ganji method). Also, other research based on the finite element method has been done^40–46^. Reddy et al. did research on buoyant convection and heat dissipation processes of hybrid nano liquid saturated in an inclined porous annulus. They found a significant impact of the magnetic field on fluid flow and thermal transport rate^47^. Swamy et al. did research about double-diffusive convective transport and entropy generation in an annular space filled with alumina-water nanoliquid. It has also been found that higher thermal and solutal performance rates with minimal loss of system energy (entropy generation) could be achieved with a shallow annulus^48^.

Pushpa et al. worked on the optimization of thermosolutal convection in a vertical porous annulus with a circular baffle. It was found that heat and mass transport can be effectively enhanced or suppressed by the appropriate choices of baffle length and location^49^.

Sankar et al. also Double-Diffusive Convection from a Discrete Heat and Solute Source in a Vertical Porous Annulus. They found that the location of heat and solute source has a profound influence on the flow pattern, heat, and mass transfer rates in the porous annulus^50^.

Pushpa et al. studied the Numerical Study of double-diffusive convection in a vertical annular enclosure with a baffle. It has been observed that the baffle size and location had a very important role in controlling the thermosolutal convective flow and the corresponding heat and mass transport characteristics^51^. Ramesh et al. published a book about Mathematical Modelling of Fluid Dynamics and Nanofluids Mathematical Modelling of Fluid Dynamics and Nanofluids^52^.

Examining the results of the FEM is the main idea of this research. This paper aims to investigate various non-dimensional parameters influencing the double diffusion natural convection in a sloped bi-layered cavity. These parameters are the Darcy number “*Da*”, Lewis number “*Le*”, Rayleigh number “*Ra*”, buoyancy ratio “*N*”, porous media width “*K*”, the ratio of thermal conductivity “*λ*^*r*^”, and cavity rotational angle “*α*”. By usage of the finite element method (FEM), dimensionless coupled, nonlinear governing equations are solved. Results are received for a wide span of the above constants. Variations of thermal and velocity fields show the obtained findings. The objective is to endorse the accuracy of the solution and the higher speed and lower cost of our computational technique (FEM) in comparison with previous research solution methods.

## The geometry of the problem

**Figure 1:**
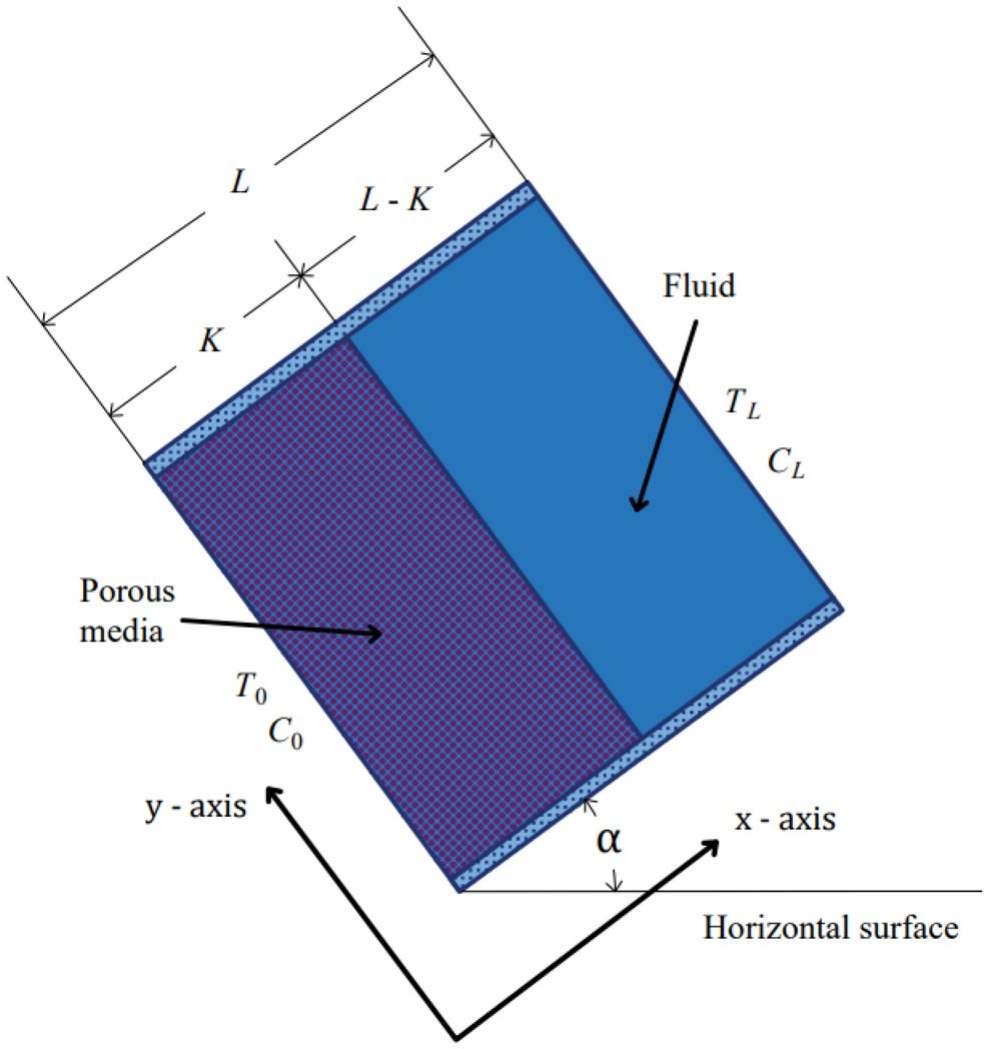
Problem geometry.

The cavity geometry is considered a 2D square with equal sides (height and width = L) depicted in Fig. 1.

The isotropic and homogeneous porous part is considered adjacent to the left wall with a thickness of “K” with values of 0.2, 0.5, and 0.8. A laminar, incompressible Newtonian fluid saturated the cavity in both layers, while the Brinkman-extended Darcy model is considered^10^.

## Mathematical formulation

Based on the geometry of the problem, saturated porous parts with different widths and an aqueous liquid are the two parallel parts that fill the enclosure space. We set different but uniform temperatures (T_0_, T_L_) and concentrations (C_0_, C_L_) on the side walls.

Continuity, momentum, and energy were the main governing equations, while the Boussinesq approximation and Brinkman-extended Darcy model were employed. As a general assumption, a 2D, laminar, incompressible flow and Newtonian fluid with constant thermo-physical characteristics is considered. The important point is a thermal equilibrium at the liquid border and the porous part. Based on the description, dimensionless parameters are presented below^34^:

1 $$(U,V) = (u,v) \cdot (L/\alpha ),$$

2 $$\theta = (T - T_{ref} )/(T_{0} - T_{L} ),$$

3 $$\varphi = (C_{0} - C_{ref} )/(C_{0} - C_{L} ),$$

4 $$(X,Y) = (x,y)/L,$$

5 $$P = pL^{2} /\left( {\frac{{\rho_{f} \cdot \alpha_{e} }}{{\varepsilon^{2} }}} \right),$$

where

6 $$T_{ref} = (T_{0} - T_{L} )/2,$$

7 $$C_{ref} = (C_{0} - C_{L} )/2.$$

At first, we use the constants below for governing equations^34^. We specify the difference between the porous layer and the cavity containing the fluid. This coefficient is applied at the beginning of the momentum equations and in the concentration equation wherever the effect of porosity should be effective:

Fluid layer:

8 $$\gamma = 0,$$

and for the porous layer:

9 $$\gamma = 1.$$

After that, by applying the dimensionless parameters in the main equations, we reach the following dimensionless equations. The continuity equation is obtained as follows^34^:

10 $$\frac{{\partial U_{i} }}{\partial X} + \frac{{\partial V_{i} }}{\partial y} = 0.$$

And X Momentum Equation:

11 $$\left[ {\frac{\gamma }{{\varepsilon_{i}^{2} }} + (1 - \gamma )} \right]\left[ {U_{i} \frac{{\partial U_{i} }}{\partial X} + V_{i} \frac{{\partial U_{i} }}{\partial Y}} \right] = - \frac{{\partial P_{i} }}{\partial X}{ - }\frac{\Pr }{{Da_{i} }}U_{i} + \Pr \cdot Rv\left( {\frac{{\partial^{2} U_{i} }}{{\partial X^{2} }} + \frac{{\partial^{2} U_{i} }}{{\partial Y^{2} }}} \right) + Ra \cdot \Pr [\theta_{i} + NS_{i} ]\sin \alpha .$$

Y Momentum Equation:

12 $$\left[ {\frac{\gamma }{{\varepsilon_{i}^{2} }} + (1 - \gamma )} \right]\left[ {U_{i} \frac{{\partial V_{i} }}{\partial X} + V_{i} \frac{{\partial V_{i} }}{\partial Y}} \right] = - \frac{{\partial P_{i} }}{\partial Y}{ - }\frac{\Pr }{{Da_{i} }}V_{i} + \Pr \cdot Rv\left( {\frac{{\partial^{2} V_{i} }}{{\partial X^{2} }} + \frac{{\partial^{2} V_{i} }}{{\partial Y^{2} }}} \right) + Ra \cdot \Pr [\theta_{i} + NS_{i} ]\cos \alpha .$$

Energy Equation:

13 $$\left[ {U_{i} \cdot \frac{{\partial \theta_{i} }}{\partial X} + V_{i} \cdot \frac{{\partial \theta_{i} }}{\partial Y}} \right] = \left( {\frac{\partial }{\partial X} \cdot \left( {\frac{{\lambda^{r} .\partial \theta_{i} }}{\partial X}} \right) + \frac{\partial }{\partial Y} \cdot \left( {\frac{{\lambda^{r} .\partial \theta_{i} }}{\partial Y}} \right)} \right).$$

And species concentration equation:

14 $$\left[ {U_{i} \cdot \frac{{\partial \varphi_{i} }}{\partial X} + V_{i} \cdot \frac{{\partial \varphi_{i} }}{\partial Y}} \right] = [\varepsilon \cdot \gamma + (1 - \gamma )] \cdot \frac{D}{Le} \cdot \left( {\frac{{\partial^{2} \varphi_{i} }}{{\partial X^{2} }} + \frac{{\partial^{2} \varphi_{i} }}{{\partial Y^{2} }}} \right).$$

Subscript “i” is the layer’s number, and for fluid particles, considered a non-slip condition on the cavity walls. In order to solve the governing equations of the problem, the boundary conditions should be applied to the walls.

For the boundary conditions of the walls, these values were considered^34^:

15 $$U,V = 0.$$

and

16 $$\theta (0,Y),\;\varphi (0,Y) = 0.5.$$

For X = 0, 0 ≥ Y ≤ 1

17 $$U,V = 0,$$

And

18 $$\theta (1,Y),\;\varphi (1,Y) = - \;0.5.$$

For X = 1, 0 ≤ Y ≤ 1

19 $$U,V = 0,$$

20 $$\left. {\frac{\partial \theta }{{\partial Y}}} \right|_{(X,0)} ,\;\left. {\frac{\partial \varphi }{{\partial Y}}} \right|_{(X,0)} = 0.$$

For Y = 0 and 0 ≤ X ≤ 1

21 $$U,V = 0,$$

22 $$\left. {\frac{\partial \theta }{{\partial Y}}} \right|_{(X,1)} ,\;\left. {\frac{\partial \varphi }{{\partial Y}}} \right|_{(X,1)} = 0.$$

For Y = 1 and 0 ≤ X ≤ 1.

We consider the continuity condition between each variable’s porous and fluid interface and heat and mass fluxes^34^.

23 $$\left. \Phi \right|_{{K^{ - } }} = \left. \Phi \right|_{{(L - K)^{ + } }} .$$

For X = K and 0 ≤ Y ≤ 1.

24 $$\left. {J\left( \Phi \right)} \right|_{{K^{ - } }} = \left. {J\left( \Phi \right)} \right|_{{(L - K)^{ + } }} .$$

For X = K and 0 ≤ Y ≤ 1.

Parameter Φ can correspond to any variable such as P, θ, φ, U, V, and J may be any heat and mass fluxes.

At interface X = K with 0 < Y < 1:

25 $$U_{1} = U_{2} ,$$

26 $$V_{1} = V_{2} ,$$

27 $$\varphi_{1} = \varphi_{2} ,$$

28 $$P_{1} = P_{2} ,$$

29 $$\theta_{1} = \theta_{2} ,$$

30 $$\frac{{\partial U_{1} }}{\partial X} = \frac{{\partial U_{2} }}{\partial X},$$

31 $$\left( {\frac{{\partial U_{1} }}{\partial Y} + \frac{{\partial V_{1} }}{\partial X}} \right) = \left( {\frac{{\partial U_{2} }}{\partial Y} + \frac{{\partial V_{2} }}{\partial X}} \right),$$

32 $$\frac{{\partial \theta_{1} }}{\partial X} = \frac{{\partial \theta_{2} }}{\partial X},$$

33 $$\frac{{\partial \varphi_{1} }}{\partial X} = \frac{{\partial \varphi_{2} }}{\partial X}.$$

The effect of pressure in the cavity is undeniable, so this issue was considered in the calculations using the following formula^53^:

34 $$\left( {\frac{{\partial^{2} p}}{{\partial x^{2} }}{ + }\frac{{\partial^{2} p}}{{\partial y^{2} }}} \right) = {\text{penalty}} \cdot \left( {\frac{du}{{dx}}{ + }\frac{dv}{{dy}}} \right).$$

This equation shows that the cavity’s horizontal and vertical pressure variations are related to the velocities differences. There is a constant “penalty”, which is used to make both sides of the correlation equation, and for this study, we considered the “penalty constant” equal to $10^{6}$. It is noticeable that the mentioned governing equations are applicable to the whole domain.

### Solution method

The Finite Element Method (FEM) operates by dividing the problem domain into smaller finite elements, each characterized by unique mathematical equations representing its shape and system behavior. This approach simplifies the solution of complex governing equations that are otherwise challenging to solve manually. FEM employs shape functions to interpolate nodal values and determine element-level solutions. In the context of this study, FEM is used as a numerical approach for solving governing equations. The general steps involved in FEM are as follows:

1. *Discretization* The problem domain is divided into smaller sub-domains known as finite elements, such as triangles or rectangles.
2. *Formulation* A set of linear algebraic equations is created for each finite element, describing how the unknown variables change over that element.
3. *Assembly* The individual linear algebraic equations from each finite element are combined to form a global system of equations that represents the entire domain of the system.
4. *Solution* The global system of equations is solved to obtain the unknown variables (e.g., velocity, temperature) at each finite element.
5. *Post-processing* The solution can be visualized and analyzed using graphical representations such as contour plots to gain insights into the velocity and temperature distribution over the entire geometry.

**Figure 2:**
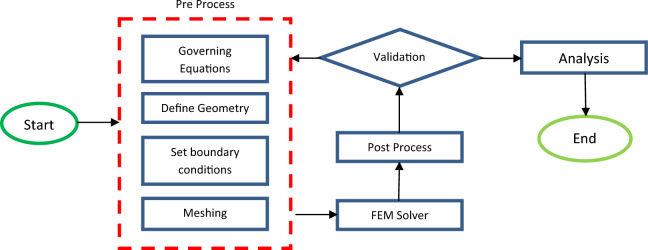
The flowchart of the numerical process.

Higher computational speed, lower computational cost, reliable results, and acceptable accuracy are the benefits of FEM. Figure 2 exhibits the flowchart of the numerical process for better understanding.

## Results and discussion

By using FEM, we obtained the following results and data. As shown in Figs. 8, 9, 10, 11, 12, 13 and 14, for dimensionless temperature $\theta$, “U” the velocity of the X direction, and “V” the velocity of the Y direction, we found effects of some significant parameters on the thermosolutal convection behavior in the chamber.

Working liquid with $Pr = 7$ and $Le = 100$ was taken for our numerical solution. Porosity is considered as $\varepsilon = 0.4$ and porous part width of K = 0.2, 0.5, 0.8 checked against $\lambda$ = 0.1, 1, 10 and rotation angle. $\alpha = 0,30,60$.

**Figure 3:**
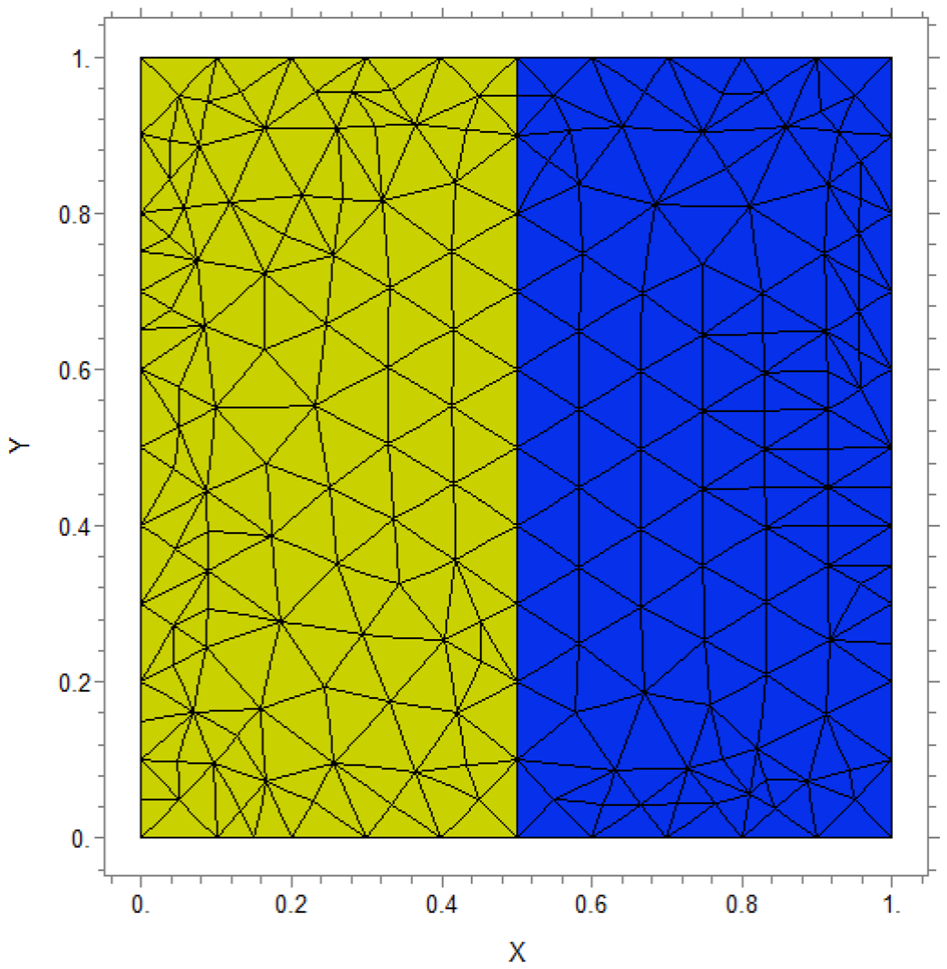
Generated mesh for a studied cavity with L = 1.

Based on Table 1 we maintain $Da = 10^{ - 4} ,Ra = 10^{6}$(thermal), and $N = - \;1$ for this problem, mass diffusivity is set as D = 1. Figure 3 shows the generated mesh in this study.

**Table 1:** Parameters values.

Parameter	Value
Porosity	$\varepsilon = 0.4$
Prandtl number	Pr = 7
Lewis number	Le = 100
Darcy number	$Da = 10^{ - 4}$
Rayleigh number	$Ra = 10^{6}$
Thermal conductivity ratio	$\lambda = 0.1,1,10$
Buoyancy ratio	N = − 1
Mass diffusivity	D = 1
Porous layer thickness	K = 0.2, 0.5, 0.8
Length width of cavity	L = 1
Cavity rotation angle	$\alpha = 0,\;30,\;60$

### Mesh study

To examine mesh independence, the values of the Nusselt numbers have been considered for various meshes.

For $Ra = 10^{6} ,\;\lambda = 1,\;Da = 10^{ - 4} ,\;N = - 1,\;Le = 100$ average Nusselt number obtained for different meshes is presented in Table 2:

**Table 2:** Mesh independent study.

Grid number	20,100	45,900	50,900	86,300
Nusselt value	4.7502	4.7519	4.7521	4.7521

According to Table 2, the mesh with a total grid of 50,900 where $Nu$ does not change significantly in the scale of $10^{ - 3}$ is the selected mesh for this research.

### Validation

**Figure 4:**
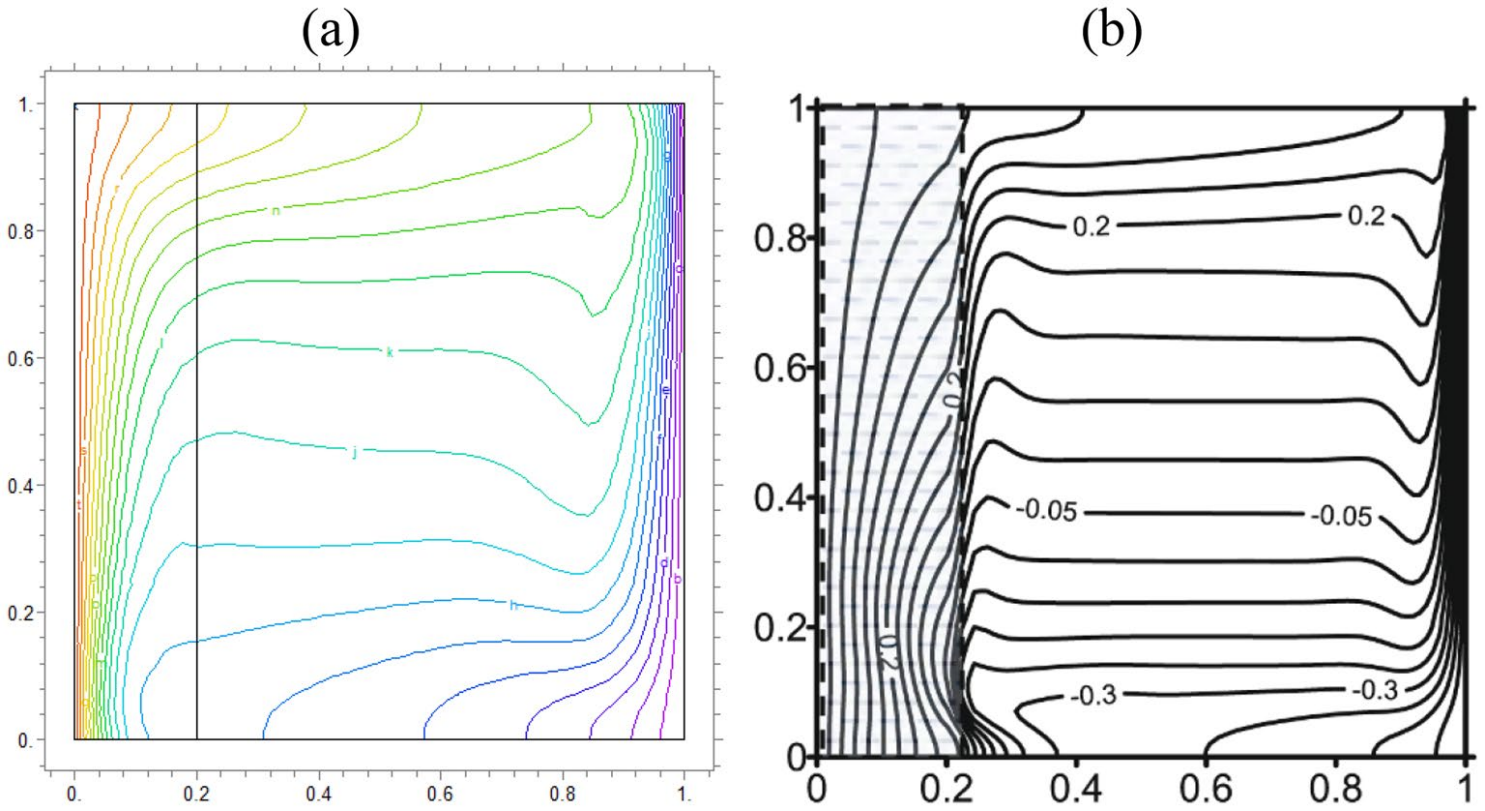
The analogy of dimensionless $\theta$ of (**a**) actual study and (**b**)^34^ for $\lambda = 0.1,\alpha = 0$ and K = 0.2

**Figure 5:**
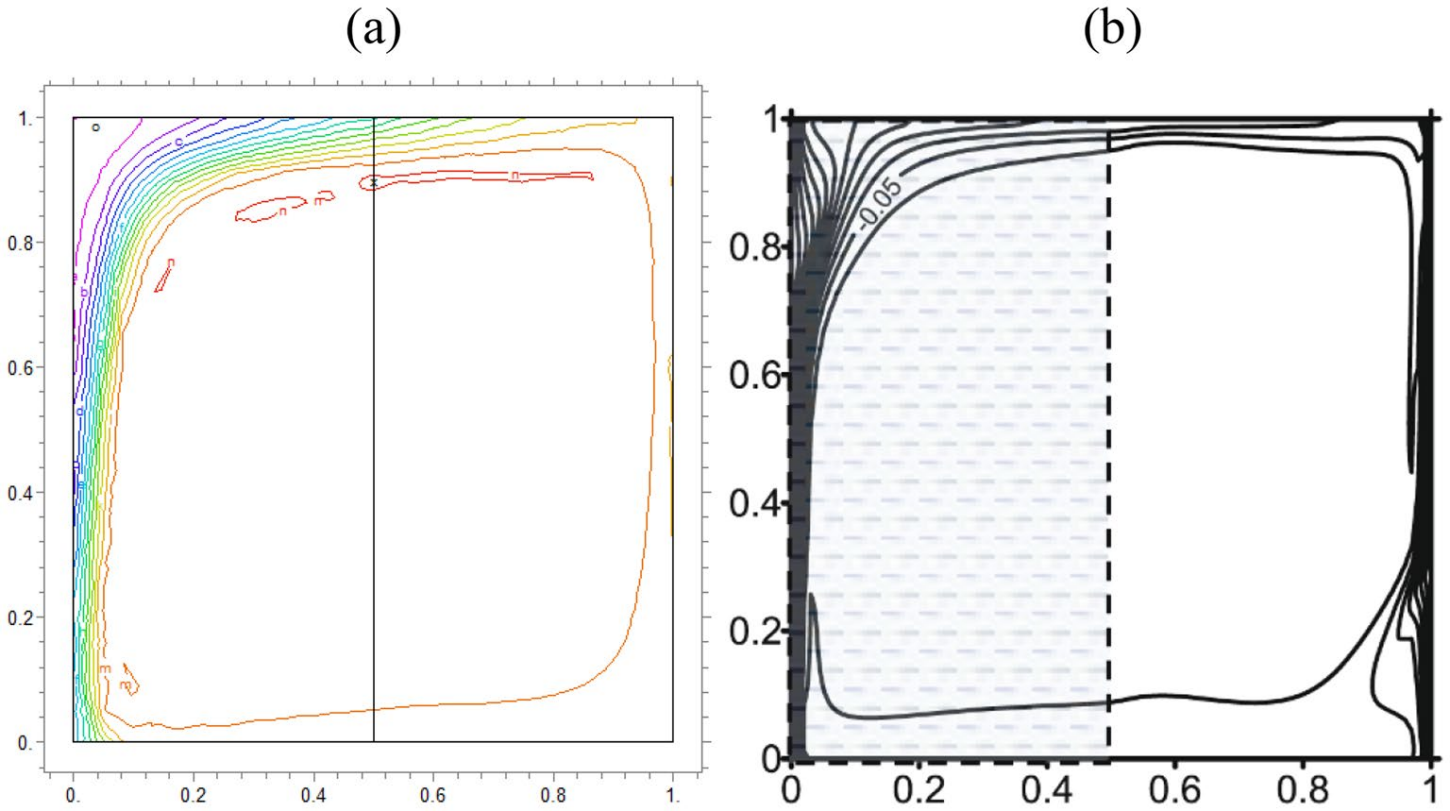
Comparison of iso-concentration lines of (**a**) present study and (**b**)^34^ for $\lambda = 1,\alpha = 30$ and K = 0.5

**Figure 6:**
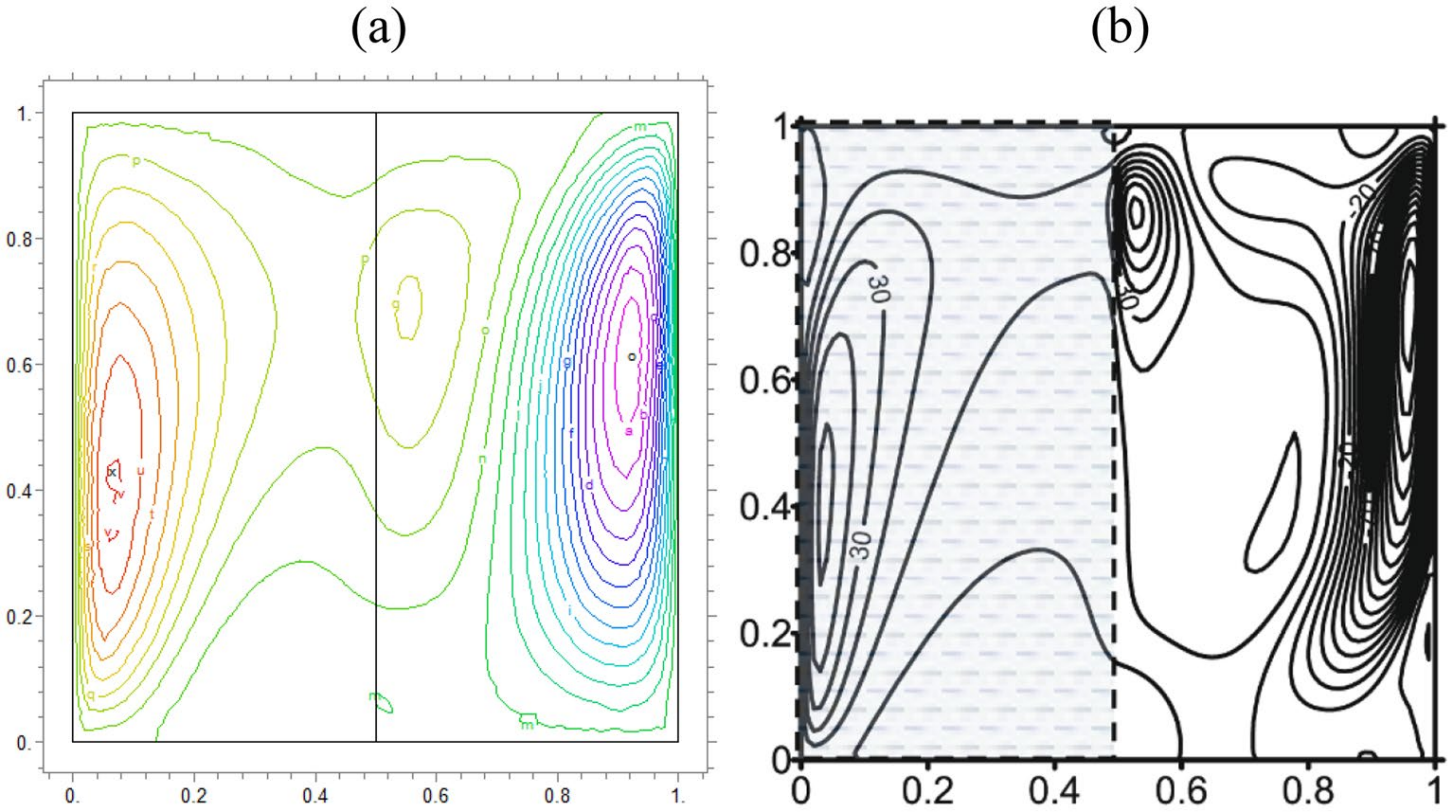
Comparison of velocity in Y direction lines of (**a**) present study and (**b**)^34^ for $\lambda = 1,\alpha = 0$ and K = 0.5

We compare concentrations, temperatures, and velocity patterns with Ref.^34^ to validate our study. Figures 4, 5 and 6 present the comparison and show a good agreement between our work with the mentioned benchmark study.

**Figure 7:**
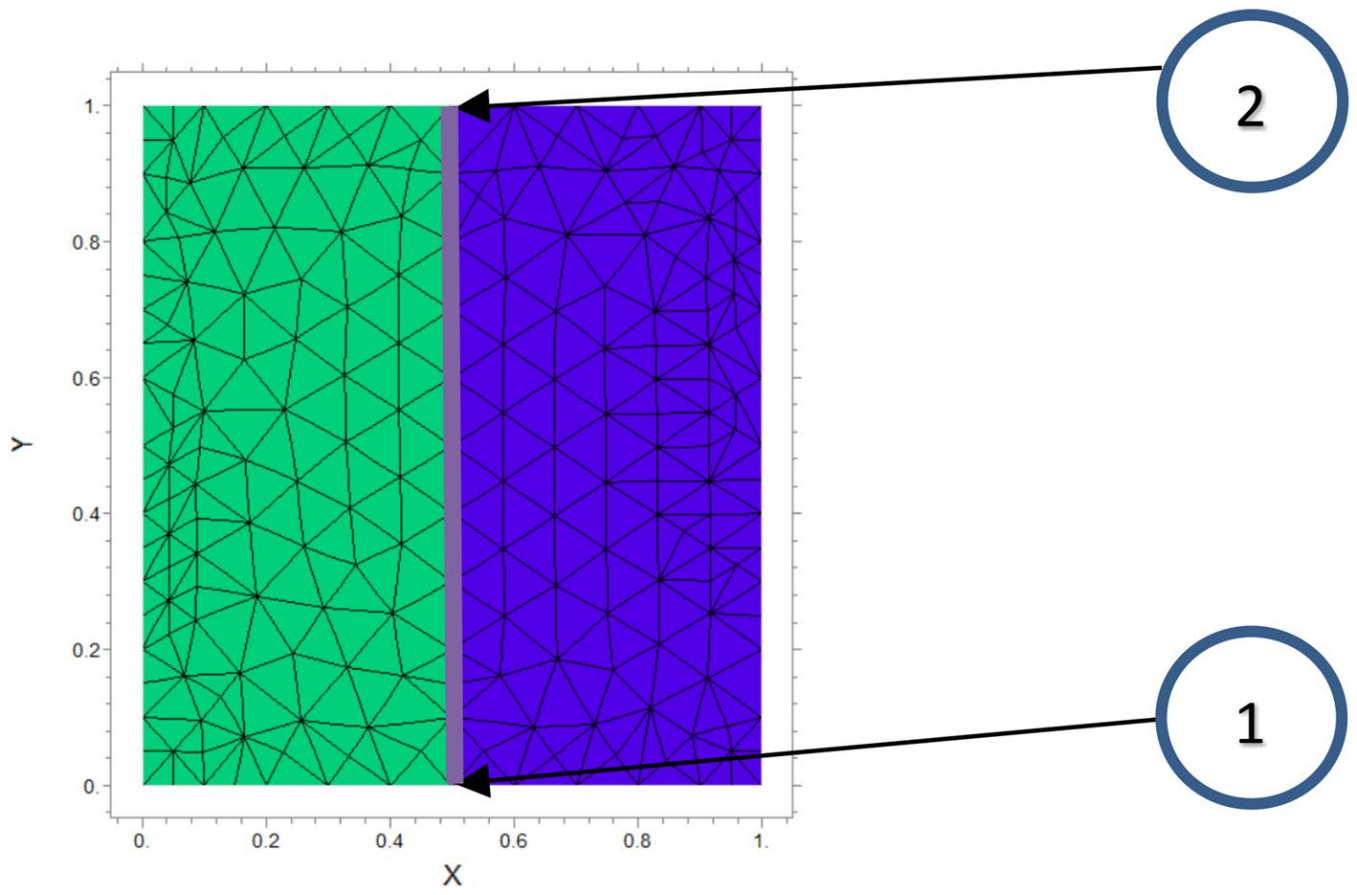
The desired hypothetical line to check the value of the average Nusselt number.

As well as patterns comparison, our obtained average $Nu$ from our calculations was 4.75, which was in good agreement with Ref.^34^. Also, the error between the obtained $Nu$ and the research^34^ is equal to err. = 0.00062, which demonstrates that the accuracy of calculations of FEM is acceptable (Fig. 7).

### Thermal distribution

**Figure 8:**
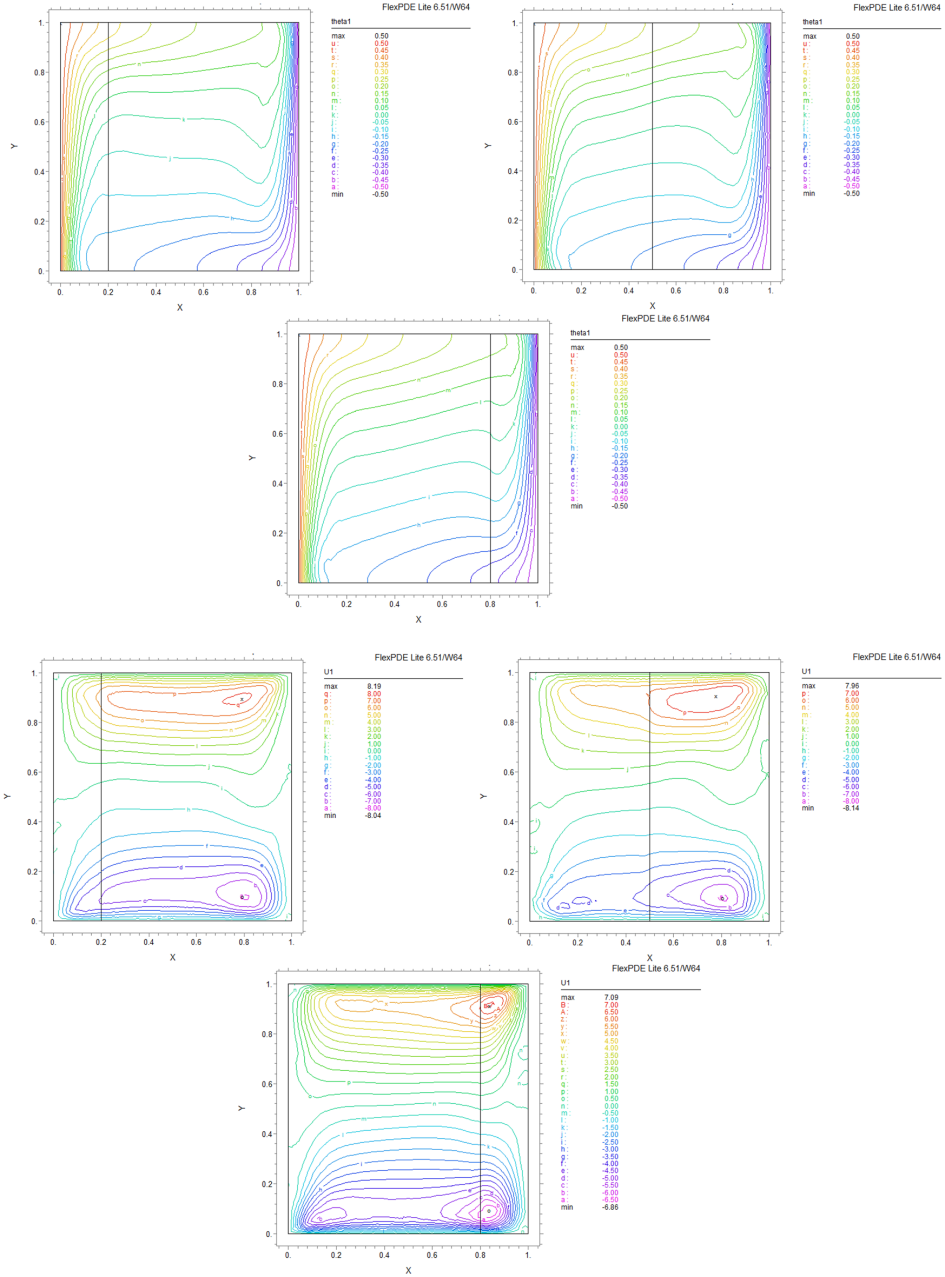

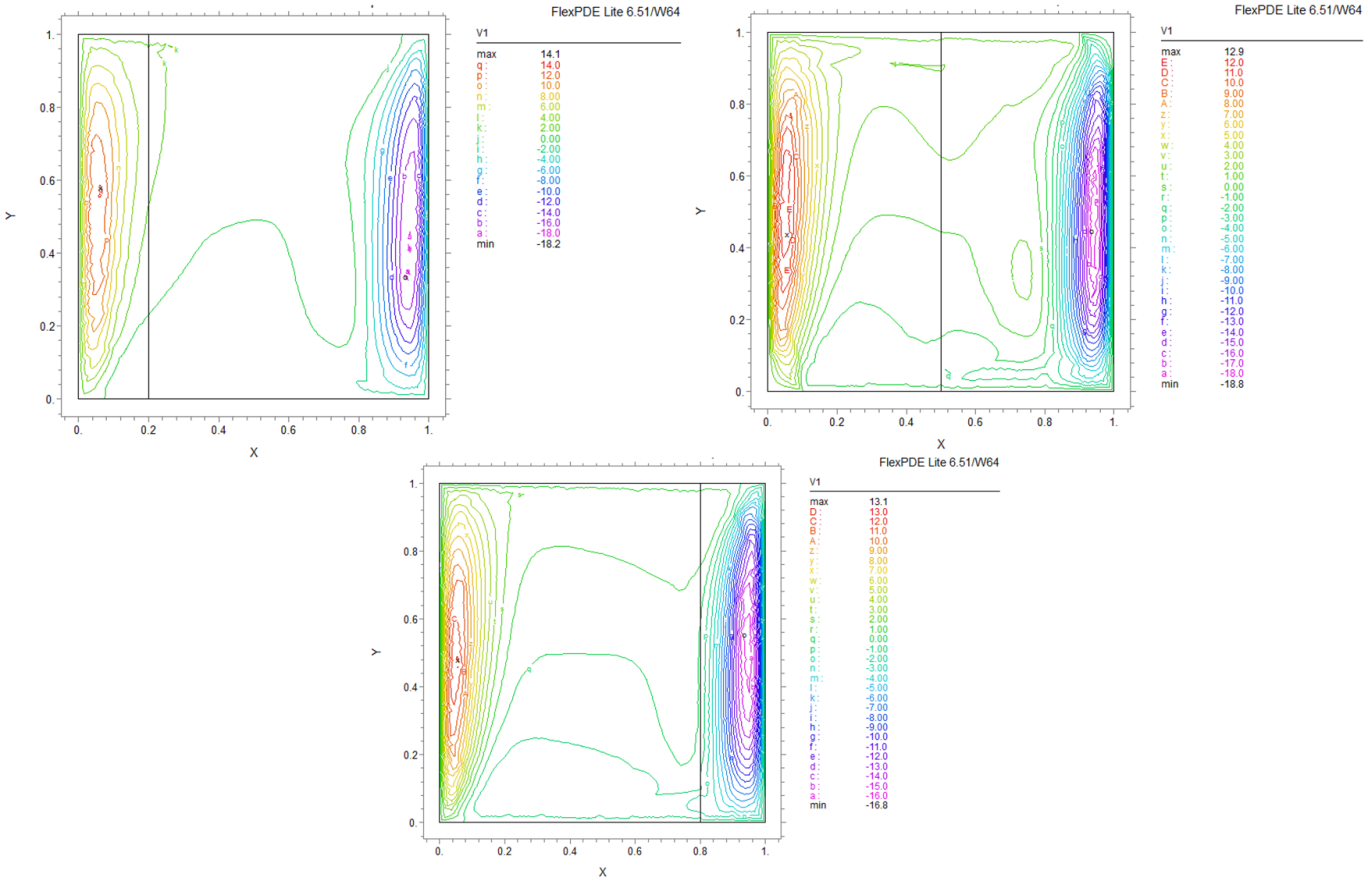
Temperature patterns and velocities (streamlines) in X and Y directions for $\lambda$ = 0.1, $\alpha = 0$ for K = 0.2, 0.5, 0.8

**Figure 9:**
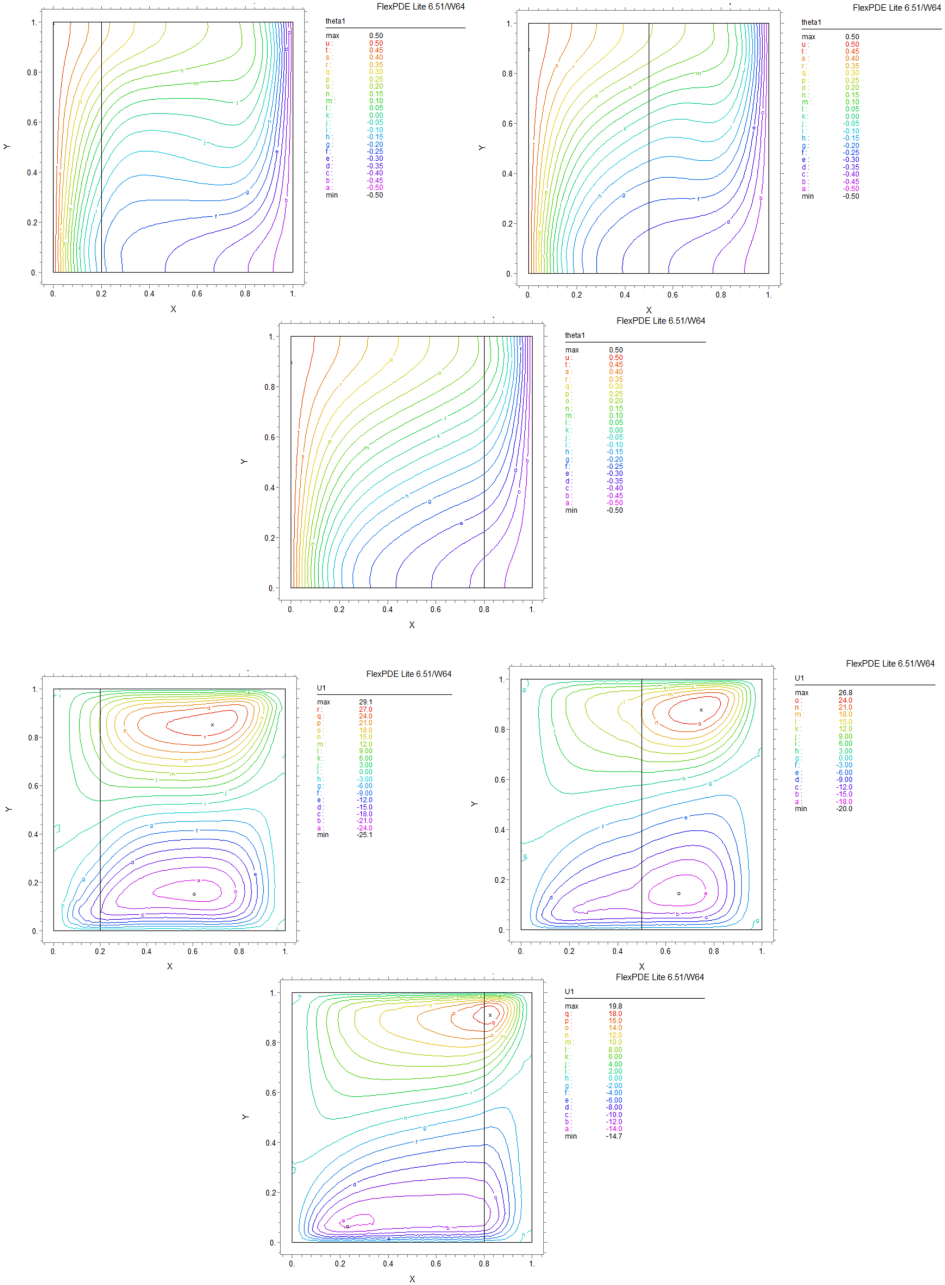

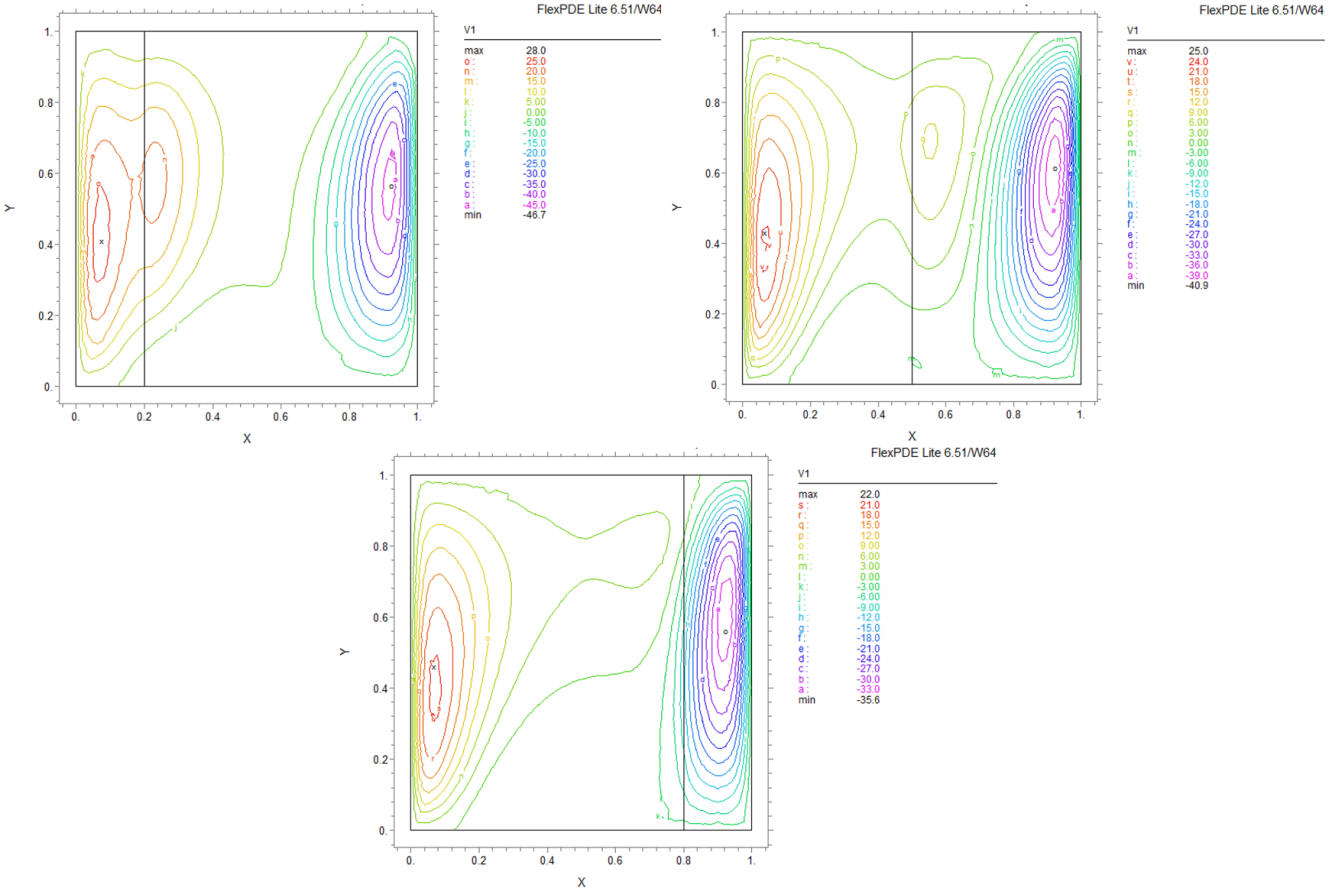
Temperature patterns and velocities (streamlines) in X and Y directions for $\lambda$ = 1, $\alpha = 0$ for K = 0.2, 0.5, 0.8.

**Figure 10:**
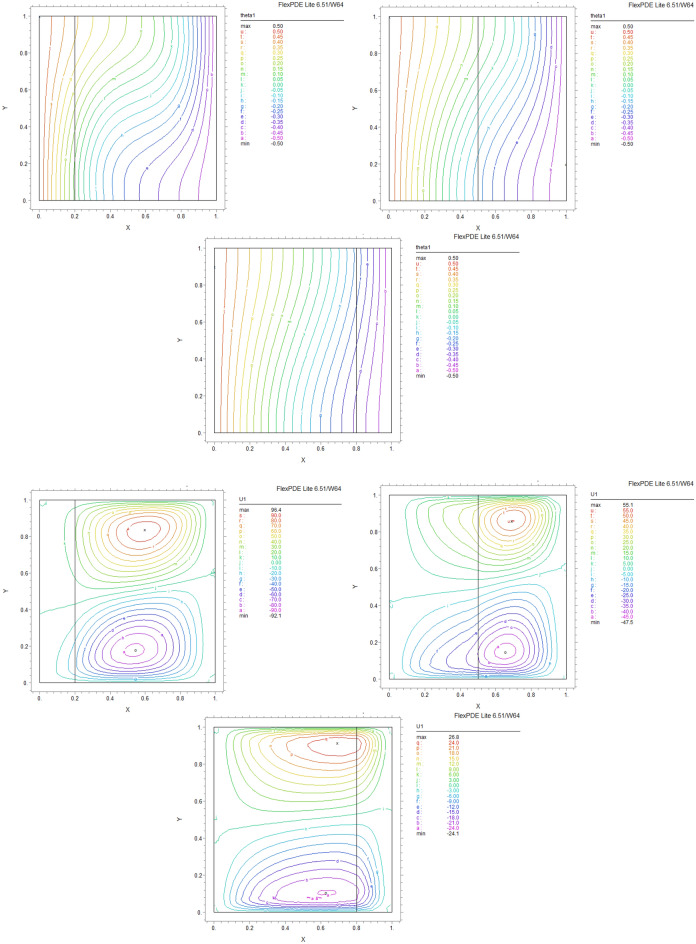

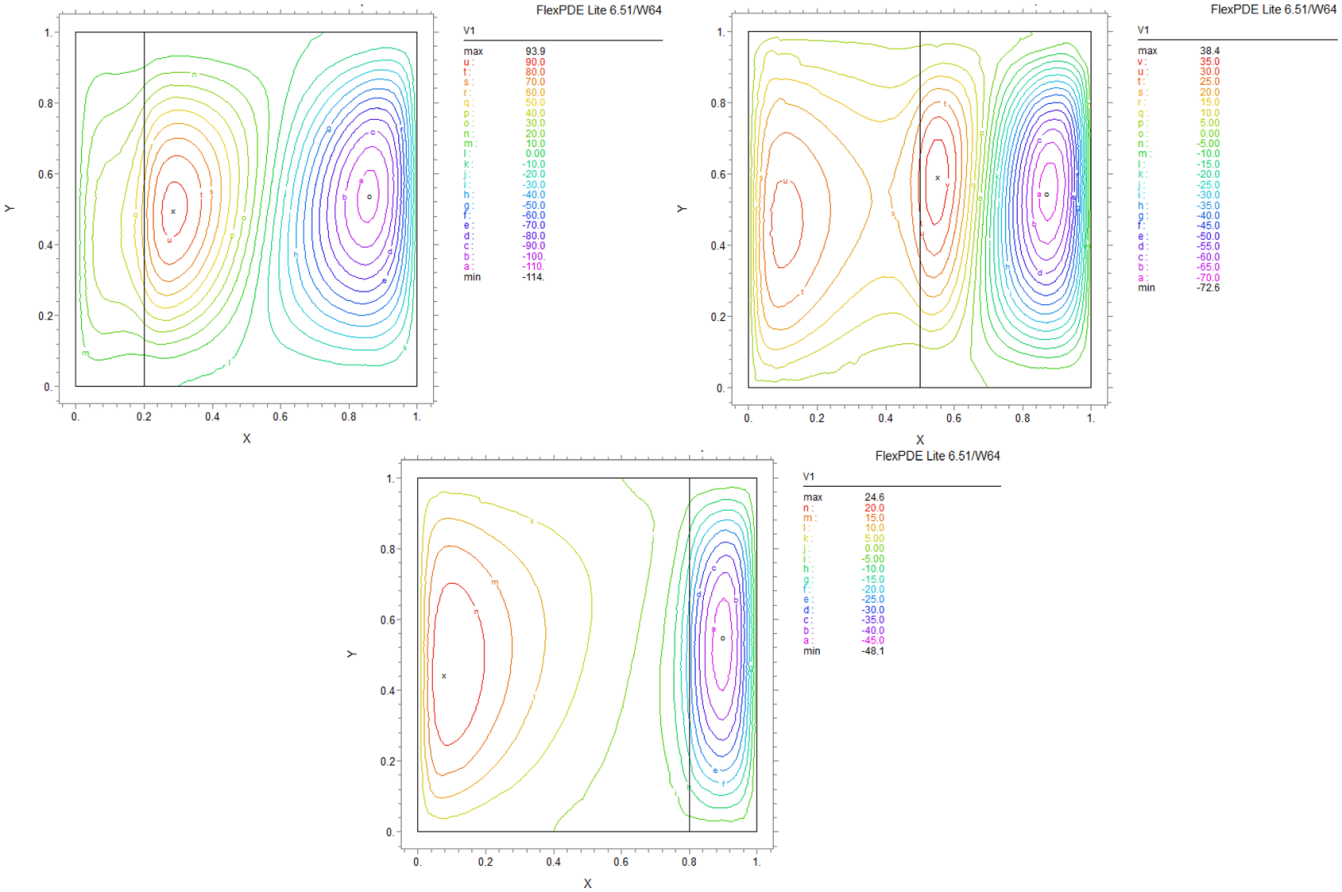
Temperature patterns and velocities (streamlines) in X and Y directions for $\lambda$ = 10, $\alpha = 0$ for K = 0.2, 0.5, 0.8

**Figure 11:**
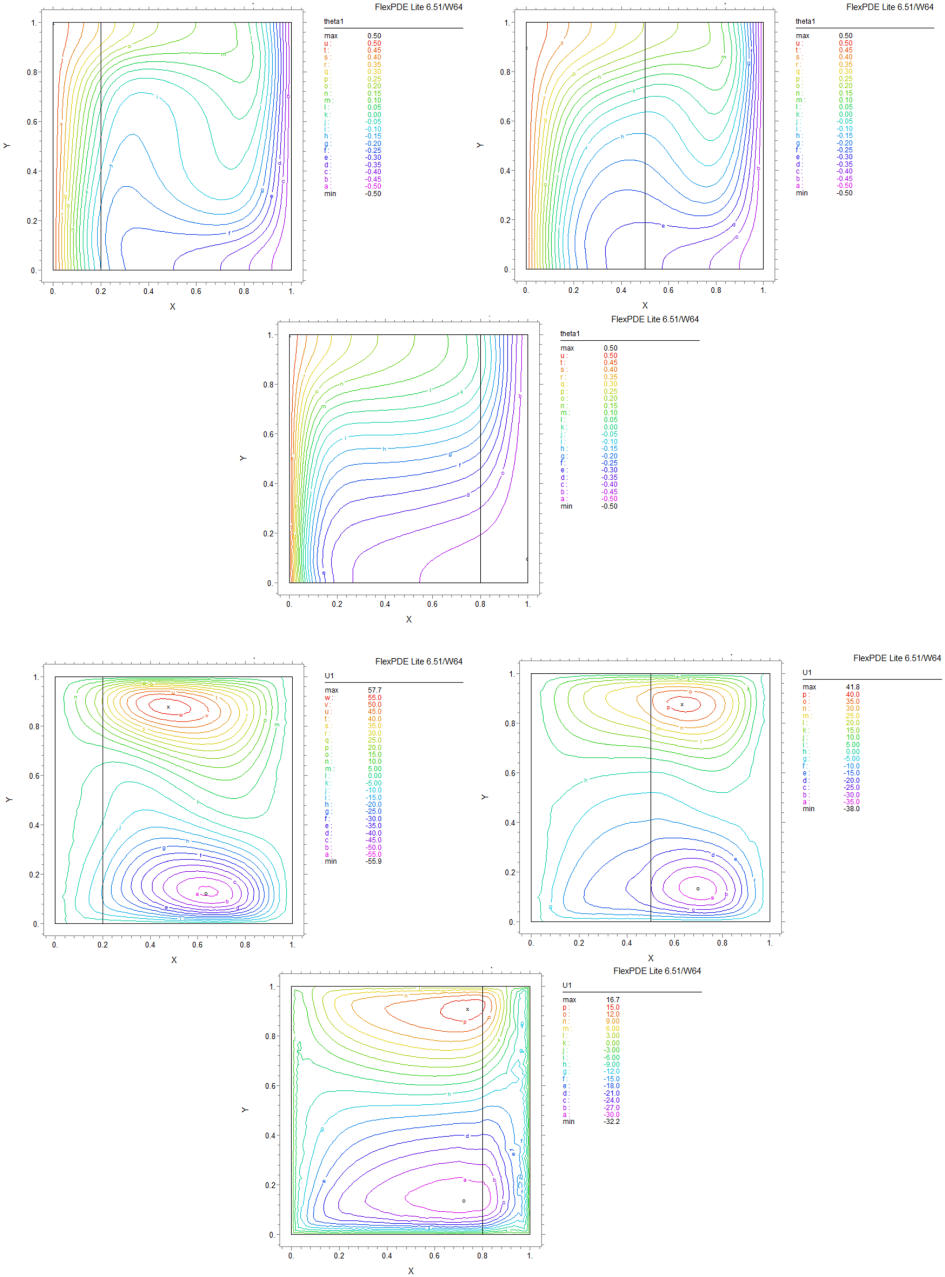

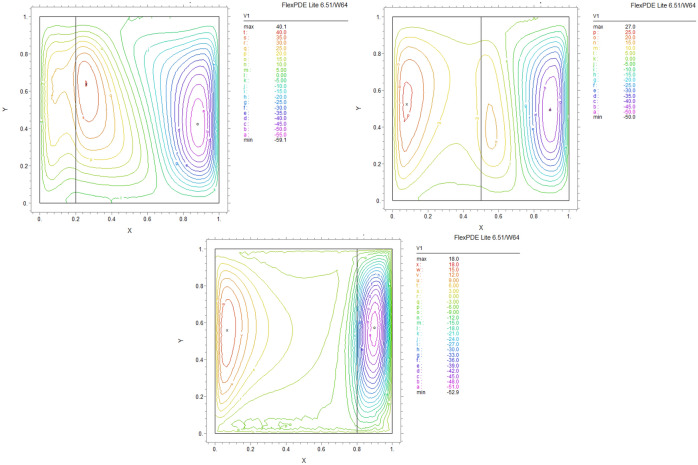
Temperature patterns and velocities (streamlines) in X and Y directions for $\lambda$ = 1, $\alpha = 60$ for K = 0.2, 0.5, 0.8

**Figure 12:**
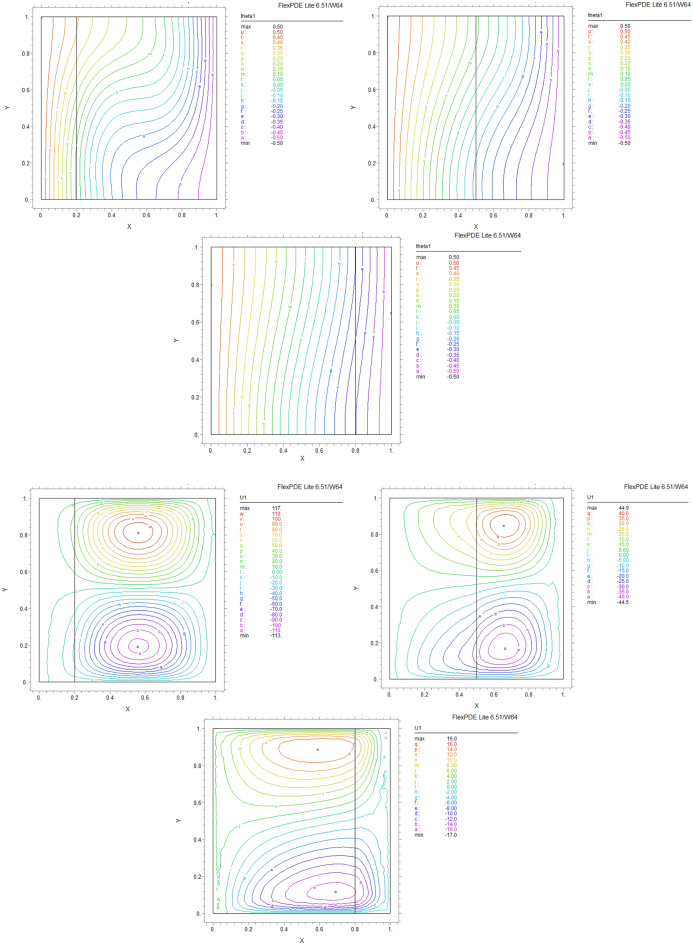

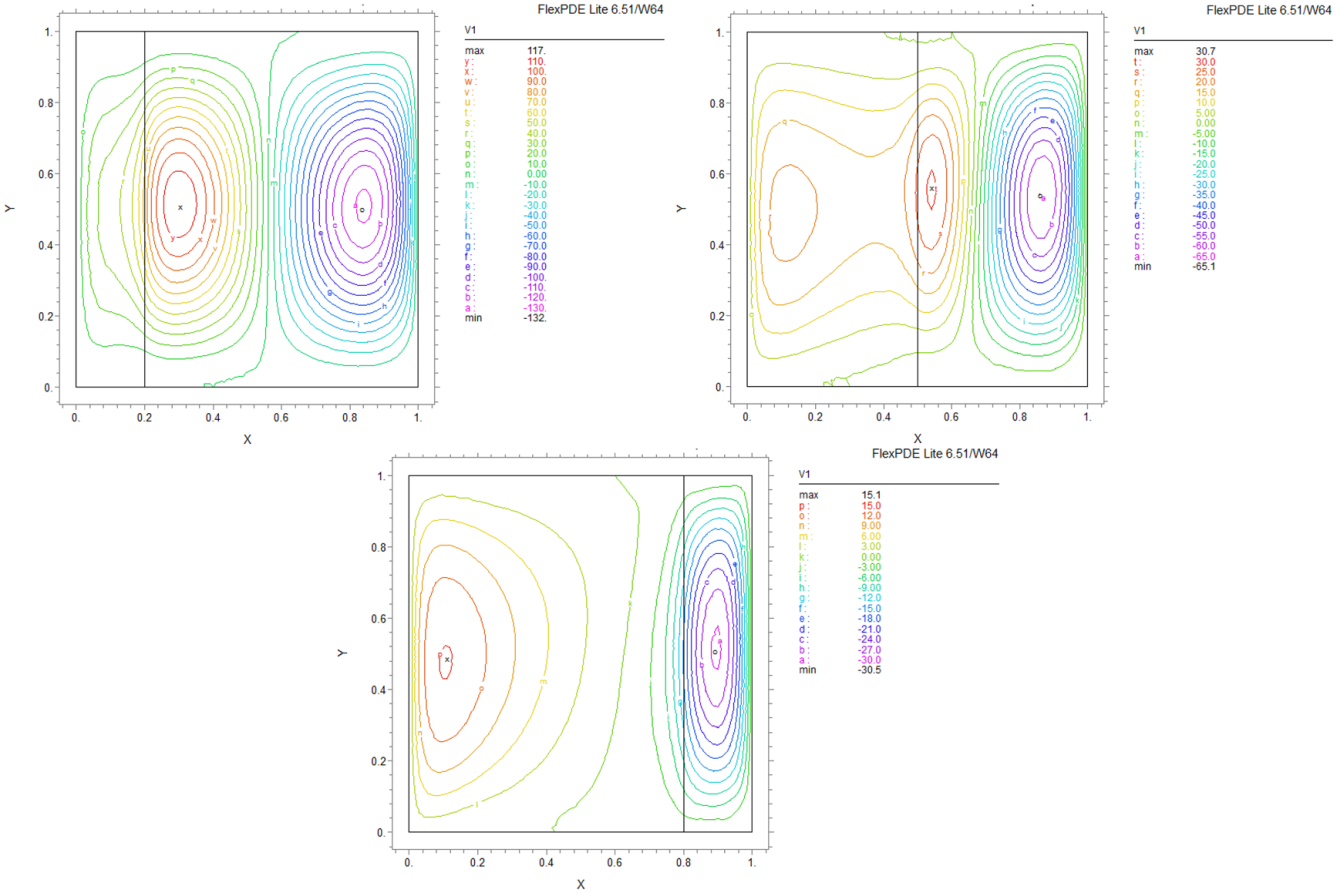
Temperature patterns and velocities (streamlines) in X and Y directions for $\lambda$ = 10, $\alpha = 60$ for K = 0.2, 0.5, 0.8

**Figure 13:**
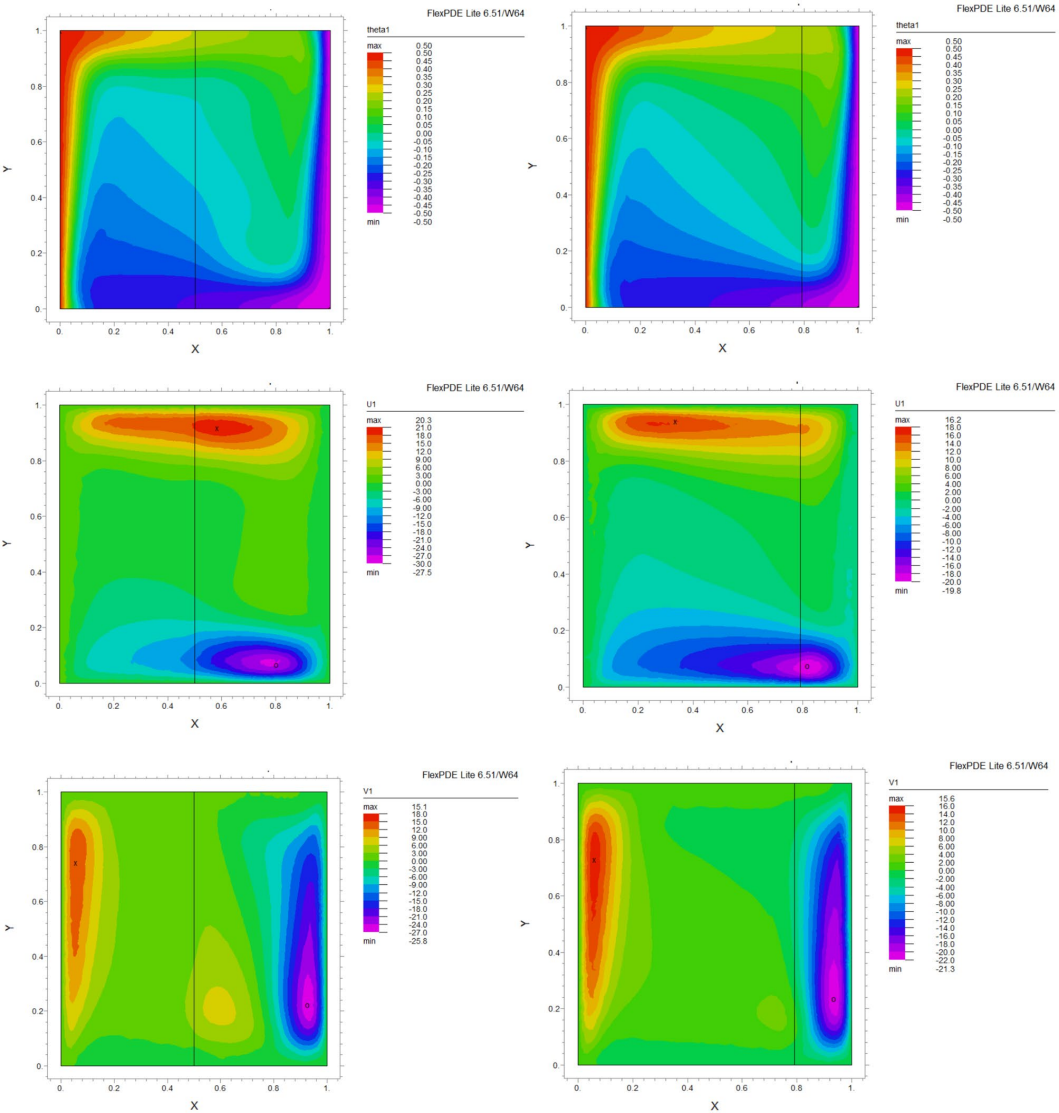
Comparison of Temperature pattern and velocities (streamlines) in X and Y directions for $\lambda$ = 0.1, $\alpha = 60$ for K = 0.5, 0.8

**Figure 14:**
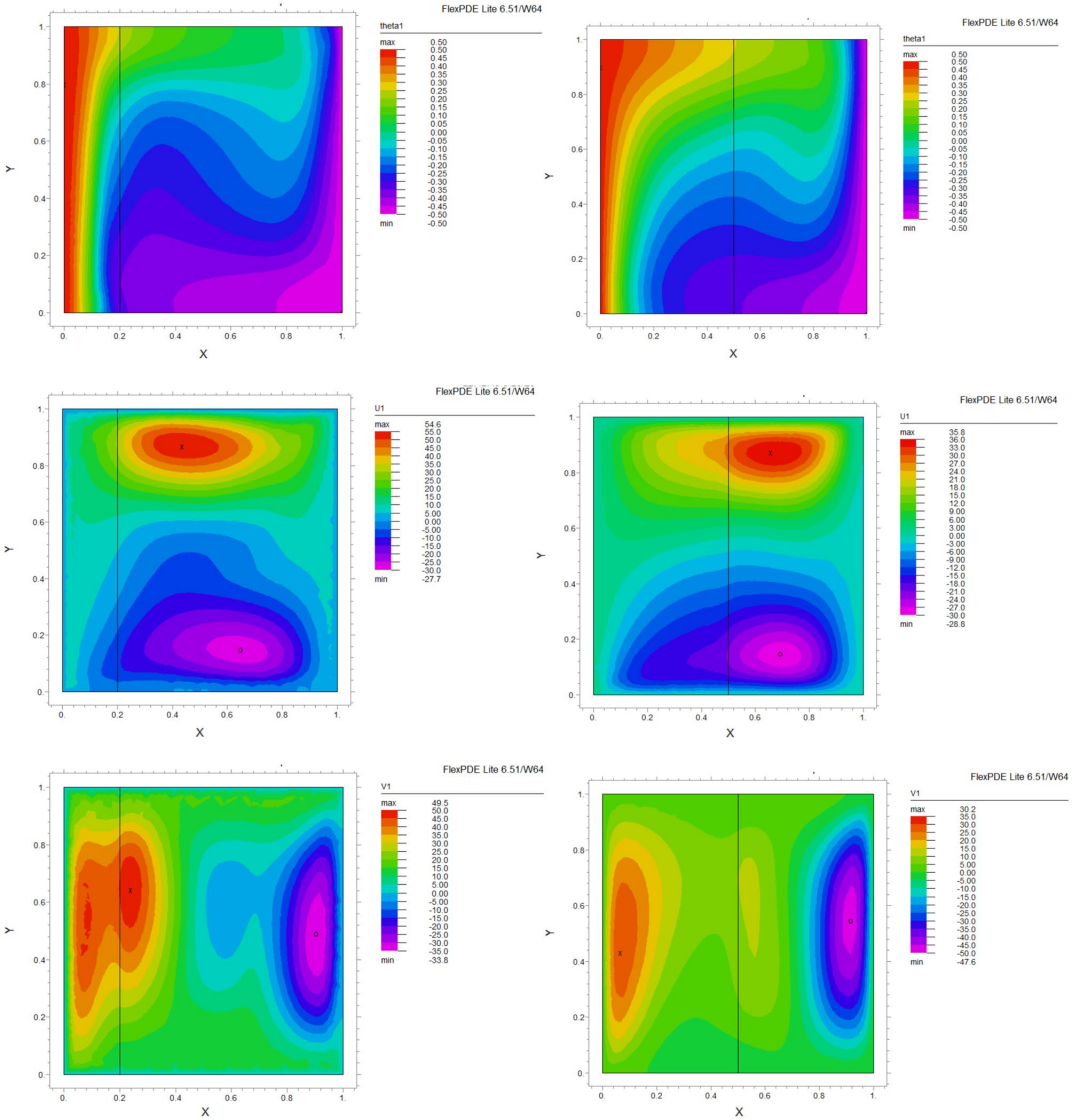
Comparison of Temperature pattern and velocities (streamlines) in X and Y directions for $\lambda$ = 1, $\alpha = 30$ for K = 0.2, 0.5

The influence of porous layer thickness K, conductivity ratio $\lambda^{r}$, and cavity inclination angle $\alpha$ on the thermal and velocities behavior are depicted in Figs. 8, 9, 10, 11, 12, 13 and 14. Our results show that the velocity vortex and eddies are formed inside the cavity. Beside, we can see thermal boundary layers adjacent to vertical walls. As we see in the pictures, thermal lines undergo a fracture after crossing the boundary between the two layers. After that, the distribution of those lines in the fluid part is parallel to the horizontal walls. With the increase of the porous layer, the isothermal lines extend towards the upper wall with a slight slope, and the distribution goes out of parallel with the horizontal walls and becomes inclined.

The results also show the parameters combined influence in porous and fluid layers by giving the different values for K and different values of $\lambda^{r}$ and $\alpha$, porous part isothermal lines raised toward the top wall and augments thermal values inside the cavity in the larger area, which specifies that the thermal exchange must be diffusive.

Various values $\lambda^{r}$ from 0.1 to 10 cause “twisted isotherms form” to change to vertical thermal lines parallel to left and right walls with uniform temperature distribution in both cavity layers. Secondly, it seems that the shape of temperature distribution goes more towards conductive heat transfer than convection. Inclination angles from 0 to 60°, influence thermal fields by turning the lines toward the lower wall. This phenomenon is more evident in the fluid layer while guiding higher thermal values to the center of the layer, as we have seen the direct impression $\lambda^{r}$ in the formulation $\theta$.

The increasing angle of the cavity causes the isothermal lines to rotate and break down and directs them toward the lower wall. This phenomenon is more evident in the “fluid-containing” part of the cavity.

Table 3 shows that the middle part of the entire cavity becomes colder as its slope increases, while on the right side of the cavity, in the only fluid section near the right vertical wall, the temperature increases with increasing angle. However, in the porous layer on the left side of the wall, the effect of increasing the angle will lead to a decrease in temperature, as in the middle part.

**Table 3:** Dimensionless temperature variations at three points of the cavity.

Rotational angle $\alpha$	Left point porous layer (x, y) (0.2, 0.5)	Right point fluid part (x, y) (0.8, 0.5)	Middle point (x, y) (0.5, 0.5)
0	0.09	− 0.12	− 0.11
30	0.008	− 0.09	− 0.16
60	− 0.02	− 0.06	− 0.17

### Velocity component field

Flow velocity lines differ in studied cases caused of the different amounts of porous part width (0.2, 0.5, 0.8). Also, remarkable changes were seen because of the variation in thermal conductivity ratios $\lambda^{r}$  = 0.1.1, 10 and the cavity rotational angle $\alpha$ (0° to 60°). As illustrated in Figs. 8, 9, 10, 11, 12, 13 and 14 different velocity vortex zones formed in the porous and fluid medium for various values of $\lambda^{r}$, K and $\alpha$. Eddies formed adjacent to lower and upper walls for “U” velocity. In contrast, for Y direction velocity, they formed adjacent to the vertical right and left walls. In some cases, secondary and third eddies are formed simultaneously in the enclosure, and the augmentation of different conductivity ratios $\lambda^{r}$ affected the formation of vortices and their behaviors, marking thermosolutal natural convection in the enclosure. The impact on the velocities is significant and encouraged in both parts with the raising of $\lambda^{r}$ different rotational angle values $\alpha$. However, increasing $\alpha$ augments “U” velocity values while the third eddy formed for “V” velocity near the upper wall and border of two layers and for bigger angle values, it moves to lower wall with smaller velocity values.

It is obvious that more porous layer thickness tends to smaller eddies and lower values of “U”. The same behavior was seen for “Y” direction velocity “V”; the third eddy disappeared in thickness K = 0.8.

The value of the maximum velocity “U” in the cavity with the thickness of the porous layer K = 0.2 is equal to U = 8.19. Meanwhile, in the thickness K = 0.8, its value is equal to U = 7.09. In this condition, there is a similar situation, and the value of the maximum speed “U” in the cavity with the thickness of the porous layer K = 0.2 is equal to U = 29.1, while in the thickness of K = 0.8, its value is equal to U = 19.8. It can be concluded that an increase in the thickness of the porous space with a porosity value of 0.40 will be accompanied by a decrease in “U” velocity, and a decrease in the velocity value leads to a decrease in the amount of thermosolutal convection.

There are larger “U” velocity values and eddies for bigger conductivity ratios $\lambda^{r}$ from 0.1 to 10. The same pattern is visible for “V” velocity, while the third eddy becomes bigger near the layer’s border while $\lambda^{r}$ increasing.

According to the results mentioned in Table 4, in different thicknesses of the porous layer, the value of the maximum velocity increases with the increase of the value $\lambda^{r}$, although the decrease of the velocity is also evident with the increase of the thickness.

**Table 4:** Variations of the maximum horizontal velocity at different porous thicknesses for different thermal conductivity ratios.

Porous layer thickness	$\lambda$ = 10	$\lambda$ = 1	$\lambda$ = 0.1
K = 0.2	96.4	29.1	8.19
K = 0.5	55.1	26.8	7.96
K = 0.8	26.8	19.8	7.09

In general, an increase $\lambda^{r}$ from 1 to a higher value of the Rayleigh number $Ra = 10^{6}$ will lead to an increase in the thermosolutal heat transfer rate and the average Nusselt number. This behavior is mostly due to the coupling of the dynamic behavior of heat and solution.

It seems that the increasing rotational angle of the cavity does not have a uniform effect on the velocity. While bigger angles result in higher velocity values, in some cases, by increasing this angle from 30 to 60°, velocities are reduced. The results show that there is a slope angle of more than 30°; after that, the reduction of the velocity values is obvious. When the angle is zero, the last term in the equation of momentum X does not exist, so the effect of Buoyancy is eliminated. As the angle increases, this effect becomes more apparent, a term highlighted in the equation of momentums.

### Conductivity ratio impact

By increasing $\lambda^{r}$ from 0.1 to 10, “twisted isotherms form”, changing to vertical thermal lines parallel to left and right walls. It was evident that the temperature distribution was uniform in both layers of the cavity. This matter prevents the transfer of higher temperatures to the fluid range; therefore, the fluid layer stays in lower temperatures. Even in $\lambda^{r} = 10$ it exhibits conduction formation.

Larger velocity values and eddies in both directions result from increasing $\lambda^{r}$, and the third eddy becomes bigger near the “layers border” in the Y direction. It maintains that augmentation in $\lambda^{r}$ value will help heat transfer in the enclosure.

### The inclination angle effect

Rotation of the cavity from 0 to 60° influences thermal fields by turning the lines towards the lower wall, as shown in Figs. 8, 9, 10, 11, 12, 13 and 14. For “U” velocity, by raising the inclination angle, eddies become smaller in the fluid layer, and the velocity lines become wider in favor of the porous layer and tend towards the upper wall in this layer. As depicted in the figures, the cavity’s rotation effect on velocity V behavior is more on the new eddy adjacent to the border of the two-layer. It shows that by increasing the rotation degree, the mentioned eddy tends to go down the cavity, and what is noticeable is the velocity magnitude increases in this location. It helps thermosolutal convection rate the entire cavity.

### Influence of the porous width

As seen in most figures width of the porous part has a remarkable impact on thermal pattern and velocity lines. Thermal lines are more vertical along the left wall inside the porous medium, and we can see the line breaks at the boundary between the two layers. Porosity increases the density of thermal lines inside the layer. On the opposite, what is clear is that the lines of velocity eddies are wider in this layer than in the fluid layer. This issue is especially evident in vertical velocity “V”, and new eddies are formed near the border. More porous layer thickness resulted in lower velocity values; therefore, even though the thermal conductivity increases by porous media, increasing the width of this part over the total area of the enclosure does not help the thermosolutal convection.

### Nusselt number

The Nusselt number indicates the rate of convection to conductive heat transfer. Therefore, a Nusselt number close to 1 means that conductive heat transfer and convection are close together. Larger Nusselt numbers indicate greater convection heat transfer and its dominance over conduction.

According to the explanation, the general formula of the Nusselt number in the cavity is equal to^23^:

35 $$Nu = \int\limits_{0}^{l} {\frac{\partial \theta }{{\partial x}}dy.}$$

Empirical formulas are presented based on the situation inside the cavity. For multi-layer cavities, these formulas are different according to the type of layer. For example, for the solid layer, the transfer of heat flux is completely due to thermal conduction, but in the fluid layer, it is convective. In porous materials, we see both types of heat transfer, depending on the porosity.

For a cavity with a porous first layer and a fluid next layer (present research), this formula is presented as follows^23^:

36 $$Nu = 0.35 \times Ra^{1/4} (\theta_{in} + 1/2)^{5/4} .$$

In this formula, “Ra” is the Rayleigh number and $\theta_{in}$ is the dimensionless temperature value at the junction of the two layers. In order to check the average Nusselt number, the values of this number on the middle line of the cavity obtained from the point (0.5, 0) to the point (0.5, 1). The desired hypothetical line is shown in Fig. 7.

#### Porous layer thickness effect on the Nusselt number

**Graph 1:**
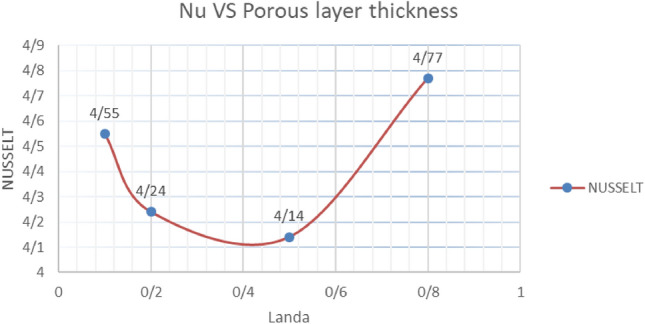
Nusselt number values against the change in the thickness of the porous layer with $\lambda = 1$ and the 0-degree slope of the cavity.

Based on Table 5, the indirect effect of increasing the thickness of the porous layer on the average Nusselt number in the mentioned hypothetical line shows that with the increase in thickness, the Nusselt number decreases, but at a higher thickness equal to 0.8, we will see an increase in the Nusselt value (Graph 1).

**Table 5:** Nusselt number values according to the change in the thickness of the porous layer $\lambda = 1$ and the 0-degree slope of the cavity.

Porous layer thickness	0.1	0.2	0.5	0.8
Nusselt	4.55	4.24	4.14	4.77

#### Thermal conductivity ratio impact on the Nusselt number

**Graph 2:**
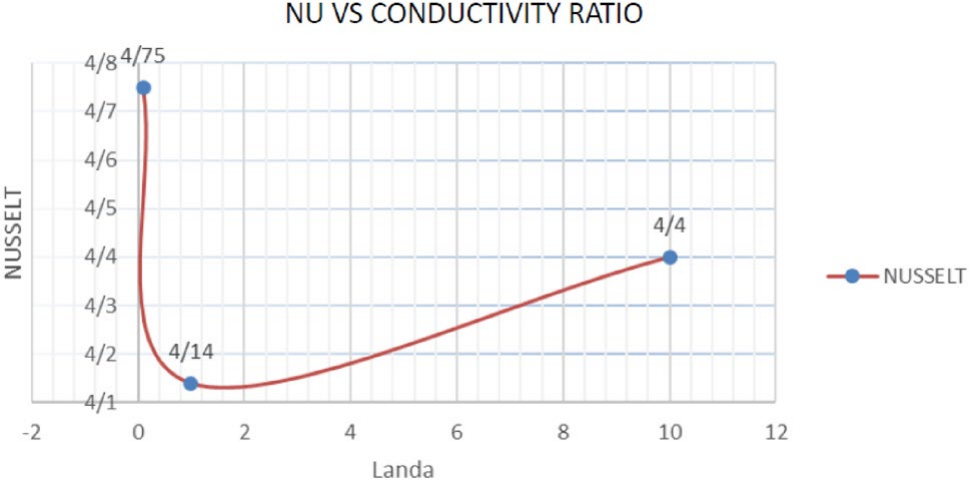
Nusselt number values against the change in the thickness Thermal conductivity ratio with k = 0.5 and the 0° slope of the cavity.

Based on the results obtained in the Table 6, increasing the thermal conductivity ratio in values less than one and from 0.1 to 1 leads to a decrease in the average Nusselt number, and with its increase from 1 to 10, the average Nusselt number increases. It seems that in values less than 1, the dominance of conductivity over thermosolutal convection is greater, and this parameter shows its effect more on thermal conductivity (because of the coefficient of thermal conductivity contained in it). However, with its increase from 1 due to the increasing effect in the dimensionless temperature formula, convection is considered dominant (Graph 2).

**Table 6:** Nusselt number values according to the change in Thermal conductivity ratio with k = 0.5 and the 0-degree slope of the cavity.

Thermal conductivity	$\lambda = 0.1$	$\lambda = 1$	$\lambda = 10$
Nusselt	4.75	4.14	4.40

#### The cavity rotation angle and the Nusselt number values

**Graph 3:**
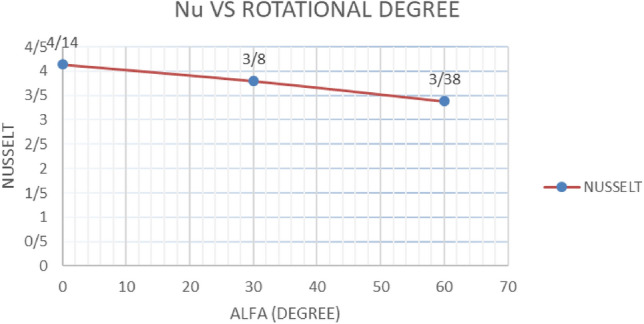
Nusselt number values against rotation angle differences.

Considering the thickness of the porous layer equal to 0.5 and the thermal conductivity ratio equal to 1, the cavity was investigated from an angle of 0 to 60°. According to the results of Table 7, increasing the angle of inclination leads to a decrease in the average Nusselt in the hypothetical line. As a result, the thermosolutal convective heat transfer is reduced, and the increase in the angle is considered a negative effect (Graph 3).

**Table 7:** Nusselt investigation for porous layer thickness equal to 0.5 and thermal conductivity ratio equal to 1 for angles 0 to 60.

Inclination angle	0	30	60
Nusselt	4.14	3.80	3.38

#### Nusselt number variations with increasing Rayleigh number

**Graph 4:**
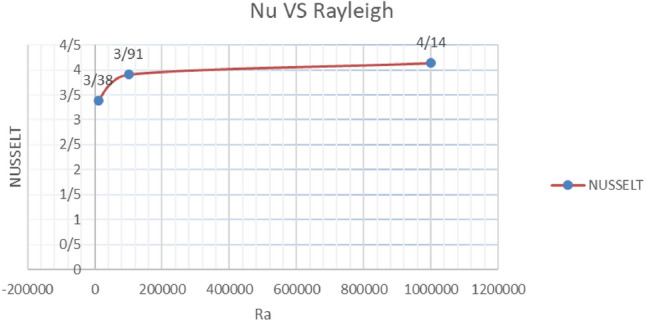
Nusselt number variations vs. Different values of Rayleigh number with $\lambda = 1,\alpha = 0,K = 0.5$

**Graph 5:**
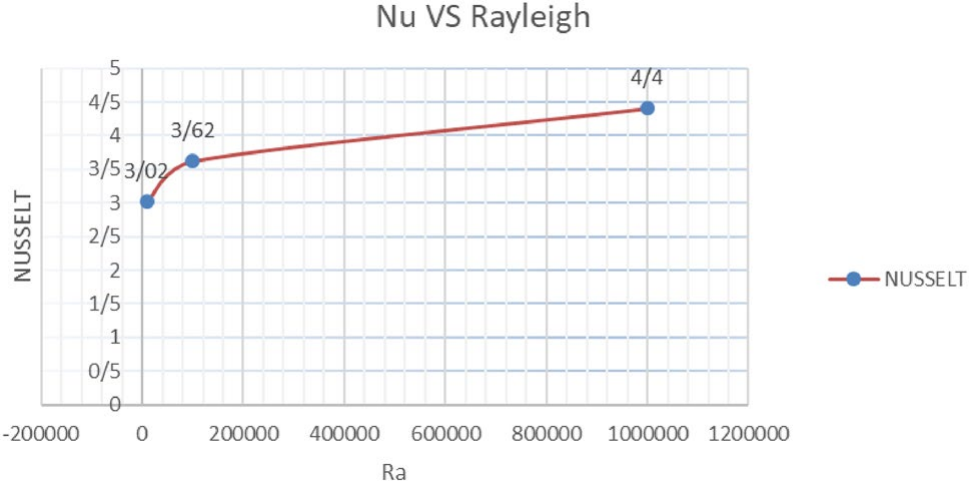
Nusselt number variations vs. Different values of Rayleigh number with $\lambda = 10,\alpha = 0,K = 0.5$

Since the Rayleigh number has a direct effect on Nusselt calculation, it is necessary to check its effect on Nusselt. Based on the results of the numerical solution of the equations, an increase in the Rayleigh number leads to an increase in the average Nusselt, which is obtained in two cases according to the Table 8, Graph 4, Table 9 and Graph 5.

**Table 8:** Average Nusselt values with increasing Rayleigh number in conditions $\lambda = 1,\alpha = 0,K = 0.5$

Rayleigh	$10^{4}$	$10^{5}$	$10^{6}$
Nusselt	3.38	3.91	4.14

**Table 9:** Average Nusselt values with increasing Rayleigh number in conditions $\lambda = 10,\alpha = 0,K = 0.5$

Rayleigh	$10^{4}$	$10^{5}$	$10^{6}$
Nusselt	3.02	3.62	4.40

The increase of the average Nusselt with the increase of the Rayleigh number is well evident. It is noteworthy that a larger Nusselt with a higher thermal conductivity ratio occurs at Rayleigh values greater than $10^{5}$. At values lower than $10^{5}$ for $\lambda = 1$, the average Nusselt value is greater than the Nusselt value of the same Rayleigh numbers for $\lambda = 10$, while this is the opposite for Rayleigh numbers greater than $10^{5}$. As a result, increasing the Rayleigh number is considered a positive effect.

#### Nusselt number variations with vs. Darcy number augmentation

**Graph 6:**
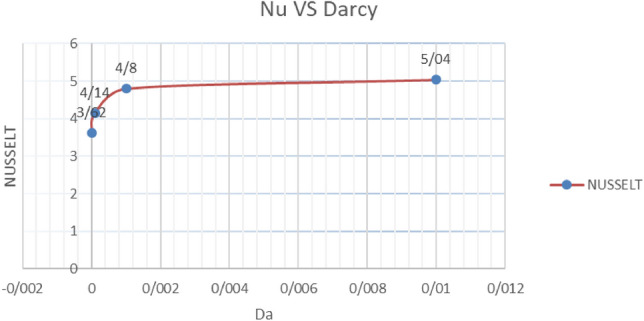
Nusselt number variations vs. Different values of Darcy number with $\lambda = 1,\alpha = 0,K = 0.5$

For Darcy numbers bigger than $10^{ - 5}$ to $10^{ - 2}$, the average Nusselt values increase as the Darcy number augments (as shown in Table 10). This issue is true for the thickness of the layers equal to 0.5 and in both cases of $\lambda = 1,\lambda = 10$, so that Nusselt reaches the maximum value of Nu = 5.04 in the Rayleigh number $10^{ - 2}$ and $\lambda = 1$ (Graph 6).

**Table 10:** Average Nusselt values with increasing Darcy number in conditions $\lambda = 1,\alpha = 0,K = 0.5$

Darcy	$10^{ - 5}$	$10^{ - 4}$	$10^{ - 3}$	$10^{ - 2}$
Nusselt	3.62	4.14	4.80	5.04

Therefore, increasing the Darcy number, which is the amount of penetration into the porous material, is considered to have a positive effect on increasing the thermosolutal convective heat transfer. The reason for that is the increase in the presence of fluid in the porous material and, naturally, the improvement of the convective heat transfer process.

## Conclusions

The manuscript presents interesting numerical results from the research regarding the distribution and behavior of the dimensionless velocities and temperature. In this study, it has been tried to solve the mentioned heat transfer with the help of a numerical method, especially the Galerkin finite element method. The solutions show the dependency of thermo-solutal parameters on convective heat transfer of square enclosure and the effects of porous medium thickness “K”, enclosure rotational angle, and thermal conductivity ratio predicted and discussed.

The important findings of this research are:

1. Porous layer thickness augmentation causes a reduction in the velocity values. It negatively affects convective heat transfer, even though it helps to increase the conduction in the porous part due to the value of its conductivity coefficient. On the contrary, this material creates a temperature gradient of the cavity’s upper surface, an increase specifically.
2. Cavity rotation from 0 to 60° influences thermal fields by turning the lines towards the lower wall. Higher temperature gradients appear in the fluid layers when $\alpha$ adopting bigger values.
3. Augmentation of U velocity values and formation of third eddy for V velocity near the upper wall and border of two layers result from increased in $\alpha$. Higher velocity values make the encouragement of heat transfer.
4. Increasing $\lambda^{r}$ from 0.1 to 10 would cause twisted form isotherms to change to vertical thermal lines with uniform temperature distribution in both fluid and porous layers among the cavity. This matter prevents the transfer of higher temperatures to the fluid range; therefore fluid layer stays in lower temperatures, specifically in $\lambda^{r}$ = 10.
5. Larger velocity values and eddies in both directions result from increasing $\lambda^{r}$, and a more considerable third eddy was shown near the border of layers in Y directions. It maintains that augmentation will help the rate of thermal transfer in the enclosure.
6. The increase in the thermal conductivity ratio of the fluid, along with the increase in the thickness of the porous layer, causes uniform and better temperature distribution in the cavity, and the warmer layers of the fluid fill half of it.
7. An increase in the thickness of the porous part will be accompanied by a decrease in the velocity of U, and it will lead to a decrease in the amount of heat transfer. Therefore, the effect of increasing the thickness is negative.
8. In different thicknesses of the porous layer, the value of the maximum velocity increases with the increase of the value $\lambda^{r}$.
9. In general, an increase $\lambda^{r}$ from 1 to a higher value in the Rayleigh number $Ra = 10^{6}$ will lead to an increase in the thermosolutal heat transfer rate and the average Nusselt number. This behavior is mostly due to the coupling of the dynamic behavior of heat and solution.
10. Increasing the inclination angle of the cavity leads to higher velocities. In some cases, by increasing this angle from 30 to 60°, we are accompanied by a decrease in velocity values. The results show that there is a critical angle of inclination in the range between 30 and 40°; after that, velocity decreases because of the significant volumetric force in both axial and vertical directions created by the cavity slope.
11. The behavior of the V velocity is the same as the U. In most cases, increasing the angle to 30° will increase the amount of V. When the cavity makes an angle of 60° with respect to the horizon, a noticeable decrease in the speed of V is observed.
12. As the thickness increases, the Nusselt number decreases, but at a higher thickness equal to 0.8, we will see a higher Nusselt number.
13. Increasing the thermal conductivity ratio from 0.1 to 1 leads to a decrease in the average Nusselt number. Also, with an augmentation thermal conductivity ratio from 1 to 10, the average Nusselt number values increase. It seems that in values less than 1, conduction prevails over thermosolutal convection, and this parameter shows its effect more on thermal conductivity (because of the thermal conductivity coefficient contained in the formula). However, with its increase from 1, due to the increasing effect in the dimensionless temperature formula, convection is considered dominant.
14. We found that, in most cases, increasing the cavity rotational angle reduces the thermosolutal convective heat transfer.
15. The increase of the average Nusselt with the increase of the Rayleigh number is well evident and the increase in Rayleigh’s number is considered a positive effect.
16. Darcy numbers augmentation is considered a positive effect in increasing the thermosolutal convective heat transfer. The reason is the increase in the presence of fluid in the porous material and, naturally, the improvement of the convective heat transfer process.
17. We found that a cavity with with porous thickness of 0.2 and $\lambda^{r} = 10$ with a rotational angle $\alpha = 30$ is the cavity with higher heat transfer performance.

## Data Availability

The datasets used and/or analyzed during the current study available from the corresponding author on reasonable request.

## List of symbols

$a$: Thermal diffusivity of the porous medium (m^2^/s)
$C$: Dimensional solute concentration (kg/m^3^)
$D$: Molecular/mass diffusivity (m^2^/s)
$Da$: Layer Darcy number
$\lambda$: Thermal conductivity of the porous layer (W/m °C)
$$N$$: Buoyancy ratio
P: Pressure (dimensionless)
*Pr*: Prandtl number
$$\varphi$$: Concentration
$$R{}_{v}$$: Relative viscosity
$${\text{Sh}}$$: Sherwood number
$$S$$: Dimensionless concentration
T: Temperature
$\gamma$: The constant of a layer definition
${\text{(u, v)}}$: Velocities component in (x, y) direction, Horizontal and vertical (m/s)
$g$: Acceleration due to gravity (m/s^2^)
${\text{K}}$: Thickness of the porosity
$${\text{L}}$$: Walls length
$$Le$$: Lewis number
$$\lambda^{r}$$: Conductivity ratio
$${\text{Ra}}$$: Rayleigh number
$$Nu_{x}$$: Nusselt (local)
${\text{Nu}}$: Nusselt number (average)
${\text{Re}} {}_{x}$: Local Reynolds number
${\text{(x, y)}}$: Horizontal and vertical alignment (m)
$$\alpha$$: Tilting angle
$$\rho$$: Density
$$\upsilon$$: Kinematics viscosity
$$\theta$$: Temperature (dimensionless)
$$\varepsilon$$: Porosity

## Subscripts

PM: Porous medium
F: Fluid
Eff: Effective
i: Layer index
